# A time-frequency analysis of the dynamics of cortical networks of sleep spindles from MEG-EEG recordings

**DOI:** 10.3389/fnins.2014.00310

**Published:** 2014-10-28

**Authors:** Younes Zerouali, Jean-Marc Lina, Zoran Sekerovic, Jonathan Godbout, Jonathan Dube, Pierre Jolicoeur, Julie Carrier

**Affiliations:** ^1^Department of Electrical Engineering, Ecole de Technologie SupérieureMontreal, QC, Canada; ^2^Centre de Recherches Mathématiques, Université de MontréalMontreal, QC, Canada; ^3^Center for Advanced Research in Sleep Medicine, Hôpital du Sacré-CoeurMontreal, QC, Canada; ^4^Department of Psychology, Université de MontréalMontreal, QC, Canada

**Keywords:** wavelet ridges, source localization, maximum entropy on the mean, phase synchrony, functional connectivity, sleep spindles

## Abstract

Sleep spindles are a hallmark of NREM sleep. They result from a widespread thalamo-cortical loop and involve synchronous cortical networks that are still poorly understood. We investigated whether brain activity during spindles can be characterized by specific patterns of functional connectivity among cortical generators. For that purpose, we developed a wavelet-based approach aimed at imaging the synchronous oscillatory cortical networks from simultaneous MEG-EEG recordings. First, we detected spindles on the EEG and extracted the corresponding frequency-locked MEG activity under the form of an analytic ridge signal in the time-frequency plane (Zerouali et al., 2013). Secondly, we performed source reconstruction of the ridge signal within the Maximum Entropy on the Mean framework (Amblard et al., 2004), yielding a robust estimate of the cortical sources producing observed oscillations. Lastly, we quantified functional connectivity among cortical sources using phase-locking values. The main innovations of this methodology are (1) to reveal the dynamic behavior of functional networks resolved in the time-frequency plane and (2) to characterize functional connectivity among MEG sources through phase interactions. We showed, for the first time, that the switch from fast to slow oscillatory mode during sleep spindles is required for the emergence of specific patterns of connectivity. Moreover, we show that earlier synchrony during spindles was associated with mainly intra-hemispheric connectivity whereas later synchrony was associated with global long-range connectivity. We propose that our methodology can be a valuable tool for studying the connectivity underlying neural processes involving sleep spindles, such as memory, plasticity or aging.

## Introduction

It is believed that the characteristic patterns of spontaneous bioelectrical activity that occur during sleep, originating either from focal cortical regions or large-scale networks, reflect essential neural processes that modify the long-term functionality of the awake brain (e.g., brain plasticity, memory enhancement, see Walker and Stickgold, 2006). Among them, sleep spindles constitute a hallmark of non-rapid-eye movement (NREM) sleep. A spindle is a transient high-amplitude oscillation seen in the electroencephalogram (EEG), typically lasting approximately 500–1500 ms within the sigma band (10–16 Hz). Sleep spindles reflect the sequential activation of the reticular and dorsal thalamic nuclei, followed by neocortical targets (Steriade et al., 1985, 1987). Early animal research pointed at hyperpolarizing potentials in thalamic reticular (RE) nucleus as the neurophysiological trigger of spindle sequences (Steriade et al., 1987). Subsequently, it was demonstrated that cortico-thalamic feedback is also crucial to initiate and terminate spindle oscillations (Destexhe et al., 1998; Golshani et al., 2001; Timofeev et al., 2001; Timofeev and Bazhenov, 2005; Bonjean et al., 2011).

Cortical synchrony is a key factor involved in sustaining spindle oscillations (Timofeev and Bazhenov, 2005). Neural modeling first suggested that cortical feedback on RE cells could result in a large-scale synchronous network of spindle oscillations over the cortex (Destexhe et al., 1998). Thalamo-cortical synchronous oscillations (12–14 Hz) were subsequently measured in situ in cats (Timofeev and Bazhenov, 2005). It was observed that termination of a spindle is characterized by desynchronization of responses between cortical and thalamocortical neurons (Steriade et al., 1998; Timofeev et al., 2001).

In EEG recordings, the mean frequency of spindles varies across the scalp. Spindles are usually slower at more anterior sites (“slower” spindles: 11–13 Hz) and typically faster at more posterior sites (“faster” spindles: 14–16 Hz; Jankel and Niedermeyer, 1985; Jobert et al., 1992). Interestingly, Andrillon et al. (2011) showed that faster spindles observed at electrode Cz emerge usually around 500 ms before the onset of slower spindles at frontal sites. The scalp topography of spindle frequency may reflect distinct neurophysiological processes (Timofeev and Chauvette, 2013). According to this suggestion, higher-frequency and earlier spindles would reflect initial thalamocotical interactions, predominant in central regions; whereas lower-frequency and later spindles would reflect secondary cortico-cortical interactions, spreading over frontal regions.

Recent studies also reported that intra-spindle frequency is not stable in time. For most spindles, the dynamics is characterized by a progressive frequency slowing, even at posterior EEG electrode sites (Schonwald et al., 2011). When analyzing separately spindles with high and low frequency, Urakami (2008) showed the shift in frequency over time is well explained with two dipolar sources located deep in the postcentral and in the precentral regions, bilaterally. However, the synchronous neural networks involved in sleep spindles, and the dynamics of their deployment over time, have never been characterized.

This article presents a new methodology to characterize the neural generators of EEG spindles from the perspective of cortical synchrony as measured on MEG. Thus, we considered frequency-locking among MEG sensors within a time window around spindles marked on the simultaneous EEG. MEG frequency-locking consists in transient synchronous events (SEs) during which activity recorded by a subset of sensors oscillate at the same frequency. There are two main reasons to consider MEG frequency-locking to understand cortical activity during EEG spindles. First, MEG recordings are spatially less corrupted with spurious correlations than EEG (absence of reference electrode, no spatial blurring from conduction on the scalp). Second, the source localization of oscillatory patterns is more tractable in MEG, where an adequate model of data generation does not involve current propagation through inhomogeneous tissues.

In the present work, we localized the cortical generators of the frequency-locked MEG events during EEG spindles. In addition we characterized for these events the cortical distribution of power and the cortico-cortical functional connectivity networks. To do such analyses in a unified framework, dedicated to transient oscillatory patterns like spindles, we developed a novel approach based on analytical (i.e., complex) time-frequency representations of the data from which the information related to synchrony was extracted. We identified the neural generators related to this information extracted from the MEG recordings for each spindle. The complex signal thus inferred on the sources has both information about power (amplitude) and phase, from which coupling between sources could be estimated. In addition, the frequency at which frequency-locking occurred allowed us to distinguish fast and slow rhythmic components within spindles.

Using this approach, our main results are: (1) Eighty percent of EEG spindles showed at least one significant MEG frequency-locked event; (2) within spindles, the central frequency of early frequency-locked activity was mainly distributed around 14 Hz (fast) whereas it is distributed around 12 Hz (slow) for late frequency-locked activity; (3) early frequency-locking, no matter its frequency, emerges mainly from parietal regions whereas late frequency-locking emerges from a much broader set of regions, localized mainly in frontal, parietal, and occipital areas; (4) overall long-range synchronization is lower for early than for late frequency-locking wheareas short-range synchronization is higher for early than for late frequency-locking; (5) the cortical network for late frequency-locking involved larger numbers of connections (particularly interhemispheric) than for early frequency-locking.

## Materials and methods

### Protocol, MEG recordings, and anatomical MRI

Brain activity of 8 healthy subjects was recorded during sleep, using simultaneous MEG and EEG for a maximum period of 90 min following a period of 26 h of sleep deprivation (to insure a good probability of sleeping in the MEG laboratory). From this group, 5 young subjects were kept in the present study (see Table 1). Recordings were conducted at the Centre de Recherche en Neuropsychologie et en Cognition (CERNEC) of Université de Montréal using a 275 channel CTF-VMS whole-head magnetometer. Subjects arrived 1 h prior to their habitual bedtime and stayed awake until 2 h after their habitual wake time. During this sleep deprivation (under a research assistant supervision) activity was limited to reading or surfing on the Internet. The protocol was approved by the ETS ethics board and by the Comité d'Ethique de la Recherche of IUGM. Written informed consent was obtained from all subjects.

**Table 1:** **Subjects' information**.

**Subject**	**Age (y)**	**Duration (mn)**	**Nbr. of EEG spindles (Cz)**	**Nbr. of MEG SEs**	**Comments**
1	25	2 × 18	28	42	
2	23	4 × 18	228	N/A	Strong dental artifact (excluded)
3	26	3 × 18	109	195	
4	24	5 × 18	13	N/A	Too few spindles (excluded)
5	54			N/A	Older subject (excluded)
6	21	4 × 18	98	190	
7	24	3 × 18	37	210	
8	22	3 × 18	85	153	

The MEG recordings were split into consecutive runs of 18 min. Sleep EEG was recorded simultaneously using 56 scalp electrodes referenced to the left mastoid with a CTF EEG system integrated with the MEG system. Electrodes were positioned using the 10–10 system. In addition, the horizontal (HEOG) and the vertical (VEOG) components of the electro-oculogram were recorded using two pairs of electrodes, one pair at the outer canthi and one pair above and below the left eye, respectively. MEG and EEG were digitized at 1200 Hz with an antialiasing low-pass filter at 300 Hz (30 dB/Octave) and a high pass filter of about 0.02 Hz. MEG signals were de-noised using the CTF [CTF MEG, Coquitlam (BC), Canada] third-order synthetic gradiometer algorithm. The EEG was manually scored for sleep stages according to standard criteria (American Academy of Sleep Medicine manual, Iber, 2007). EEG spindle detection was performed visually on Cz by an experienced sleep technician. A sleep spindle was detected when a burst of oscillatory brain activity (12–14 Hz) was visible on NREM EEG for at least 0.5 using band-passed filter (1–30 Hz) (Rechtschaffen and Kales, 1968). A high resolution anatomical T1-weighted MRI scan was acquired at the Unité de Neuroimagerie fonctionelle de l'Institut Universitaire de Gériatrie de Montréal using a T1-weighted 3D MPRAGE Fast sequence (slab: 160, voxel size: 1.0 × 1.0 × 1.2 mm, TR/TE: 2300/2.94 ms, TI: 900, FOV: 256) acquired in a 3T Siemens MAGNETOM Trio scanner (Siemens Medical Solutions, Malvern (PA), USA). A mesh representation of the white/gray matter interface with 8000 vertices (sources) was extracted from the MRI scan for each subject using Brainvisa (Cointepas et al., 2001). The spatial resolution of the mesh was 5.5 ± 2.8 mm and the orientation of the sources was constrained to be normal to the surface. The forward model *G* (see Section Imaging Cortical Synchrony) that was used for the source localization was obtained from a spherical head model computed using Brainstorm (Tadel et al., 2011).

### Wavelet analysis

We consider the continuous wavelet representation of the multivariate data *M*(*t*),

(1) $$w^{\left(m\right)}\left(a,b\right)=\int_{-\infty }^{+\infty }M\left(t\right)\;\bar{\Psi_{\text{ab}}\left(\text{t}\right)}\text{ dt}$$

with the wavelet defined as usual as

(2) $$\Psi_{\text{ab}}\left(t\right)=\frac{1}{\sqrt{\text{a}}}\Psi \left(\frac{t-b}{a}\right)$$

where Ψ(*t*) is a complex valued analytical wavelet of the Morse type (see Appendix II). Ψ*_ab_*(*t*) is a short time oscillatory function scaled by factor *a* and translated in time by *b* samples. Each wavelet coefficient *w*^(*m*)^(*a, b*), where *m* refers to the data space, thus describes the oscillatory behavior of the signals *M*(*t*) at scale *a* and around time sample *b*. The scaling factor *a* was spaced along 256 scales, thus yielding a spectral resolution of ≈0.4 Hz in the sigma band. It is noteworthy that this signal representation is highly redundant and neighboring wavelet coefficients are correlated. The next section describes how we can retrieve frequency-locking information from such a redundant representation.

### Frequency-locking in the sensors space

**Figure 1:**
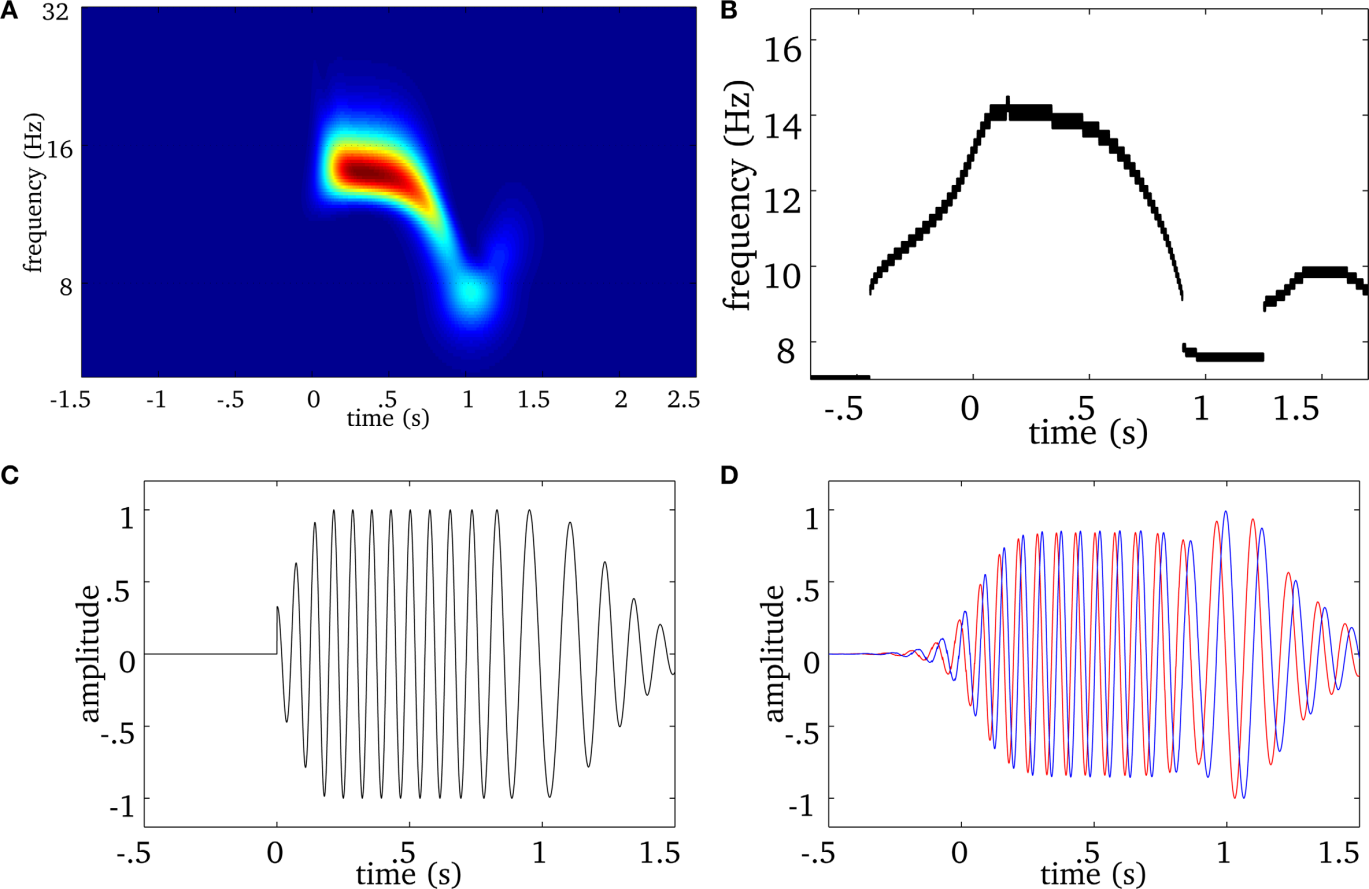
**Example of a wavelet ridge on a simulated spindle. (A)** Time-frequency plot showing the power estimated from the output of the wavelet transform of the spindle in **(C)**. **(B)** Ridges extracted from the time-frequency plot in **(A)**. **(C)** Simulated spindle oscillation. **(D)** Reconstructed real (blue) and imaginary (red) signal based on the ridge information in **(B)**. The real part of extracted ridge signal closely approximates the original signal shown in **(C)**.

From a signal representation in the time-frequency (t-f) plane, one can extract the instantaneous frequency by computing wavelet ridges (Mallat, 2008). The procedure for a univariate signal is illustrated in Figure 1. At each time sample *b*, we locate on the wavelet scalogram (Figure 1A) the local maxima in amplitude (i.e., the energy). The frequency of such maxima defines the instantaneous frequency of one oscillator present in the signal. Contiguous maxima along time are then chained into “ridge lines” *a* = *r*(*b*). The location of all ridge lines in the t-f plane is called a “ridge map” (Figure 1B) which is a binary representation of the oscillatory modes present in the signal (Delprat et al., 1994). As illustrated in Figure 1 with a simulated spindle, the complex wavelet signal (Figure 1D) along the ridge line (Figure 1B) mostly reproduces (real part shown on Supplementary Figure 1), the original oscillatory signal (Figure 1C).

**Figure 2:**
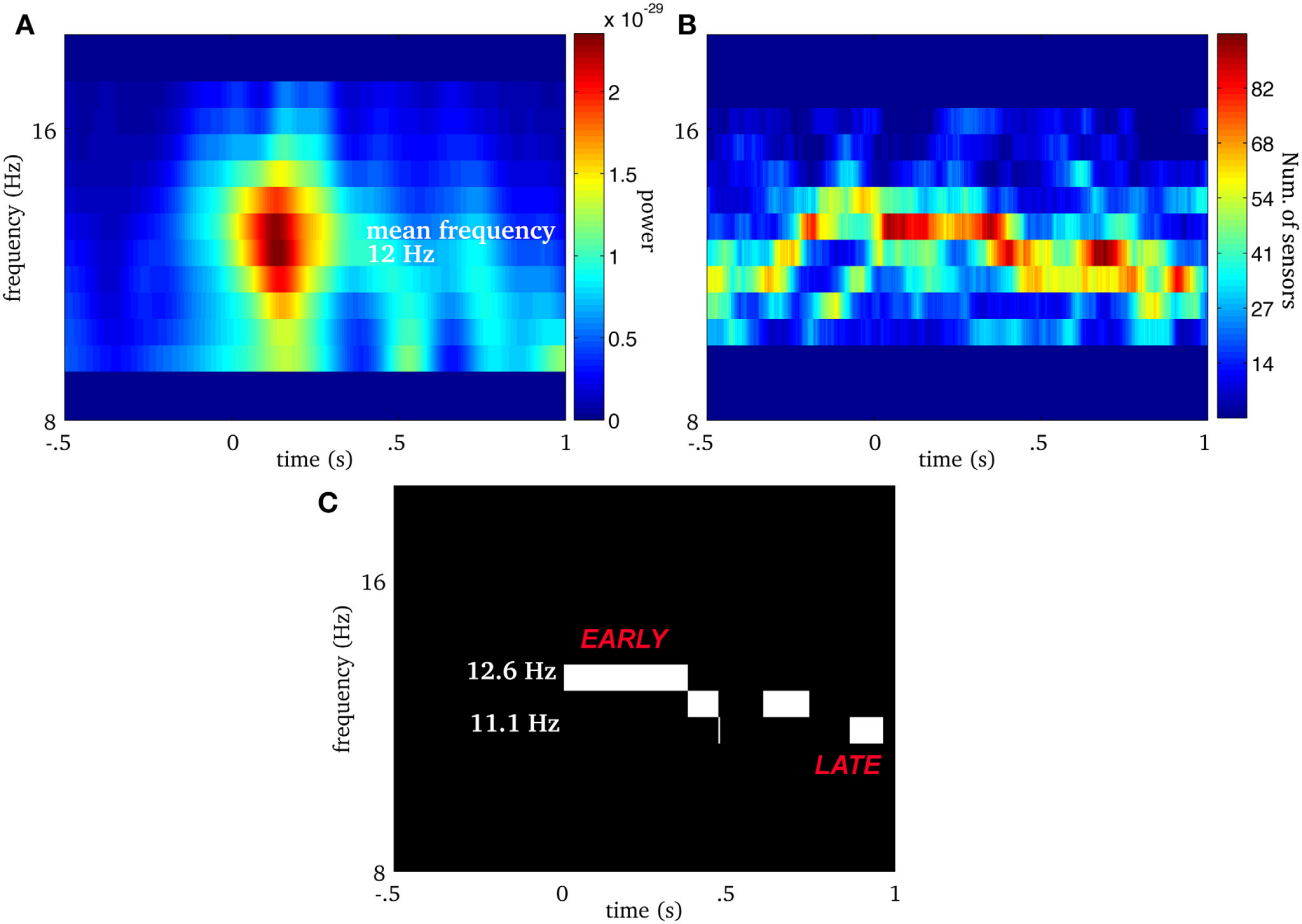
**Real spindle: (A) average wavelet power over all MEG sensors**. The EEG onset is at time equal to 0. For the same spindle, **(B)** is the multivariate ridge map obtained by summing the individual ridge maps over all MEG sensors. The colors indicate the number of sensors frequency-locked at a particular time-frequency point. **(C)** Displays multivariate ridge mask produced after data-driven thresholding of the multivariate ridge plane **(B)**. The mean power of this spindle is 12 Hz but the multivariate ridges **(B)** show synchrony above this value and even before the EEG onset (*t* = 0). In this particular case, we observe 3 multivariate ridge lines during the spindle (the discontinuity along the frequency axis reflects the limit in spectral resolution of the decomposition), with frequency starting around 12.6 Hz (early event) and ending at 11.13 Hz (late event).

We extend this approach to multivariate (i.e., multichannel) MEG signals as illustrated in Figure 2. We first compute the ridge map of each sensor (Figure 2A), then we sum them to obtain a “multivariate ridge map” (Figure 2B), the values of which reflect the number of sensors sharing common local maxima, i.e., instantaneous frequencies. On the multivariate ridge map, we track common oscillatory modes as multivariate ridge curves *a* = *r*^(*m*)^(*t*). Each curve may vary in frequency over time and reflects an episode of frequency locking among sensors. From now on, the term ‘ridge’ refers to a multivariate ridge curve *a* = *r*^(*m*)^(*t*).

### Statistically significant frequency-locking

We now define the strength of a ridge as the time average of the number of frequency-locked sensors at each time sample of the ridge. To define the minimal strength for a ridge to be considered as a spindle specific synchronous event, we define a thresholding procedure based on the rationale that synchrony must be stronger during a spindle than during baseline activity. We thus detect ridges (*r*^(*b*)^(*t*)) during a baseline window preceding a spindle (−1.5 to −0.5 s with respect to the marker) and compute their strength. Using a FDR approach, we build a cumulative distribution of ridge strength during baseline and set the cutoff such that *p* ≤ 0.05. Ridge strength cutoff is determined for each spindle, and only ridges above the cutoff are considered as “synchronous events” (SE).

### Non-linear filtering of MEG signals

Spindles typically exhibit a succession of synchronous events SEs, the first and last of which are termed respectively *early* and *late* SE (see Figure 2C). For each of these events—indexed by r, we construct an analytic ridge signal *w*^(*m*)^_*r*_(*t*)- m stands for multivariate—that consists in the complex wavelet coefficients of *all* N_s_ sensors at frequencies along the line *a* = *r*^(*m*)^(*t*):

(3) $$w_{r}^{\left(m\right)}(t)=w^{\left(m\right)}(t,r^{\left(m\right)}\left(t\right))$$

This ridge signal over the whole set of sensors is complex-valued and only exists during periods of frequency-locking between a subset of sensors. *w*^(*m*)^_*r*_(*t*) is an oscillatory component of *M*(*t*) of the form *w*^(*m*)^_*r*_(*t*) = *A*(*t*)*e*^*i* ϕ(*t*)^, where ϕ(*t*) is the instantaneous phase (Zerouali et al., 2013). This approach is analogous to the Hilbert-Huang Transform (HHT), which computes the instantaneous phase of empirical modes of the data. However, although it can successfully separate brain rhythms from EEG recordings (Bajaj and Pachori, 2012), the HHT is not readily usable for extracting synchronous components. It is noteworthy that the number of SEs that can be extracted from *M*(*t*) can vary and even be null if underlying neural generators are all asynchronous. We treat each spindle as a distinct event and quantify 4 characteristics of the SEs on a spindle-by-spindle basis: (1) the presence or absence of SEs, (2) the number of SEs, (3) the summed duration of the SEs, and (4) the onset time of the first SE.

### Imaging cortical synchrony

Given a ridge signal *w*^(*m*)^_*r*_(*t*) of length *T_r_*, we localize its cortical generators by solving the inverse problem associated with the following linear but ill-posed generative model:

(4) $$w_{r}^{\left(m\right)}\left(t\right)=G\;w_{r}^{\left(q\right)}\left(t\right)+\varepsilon_{r}(t)$$

where *w*^(*q*)^_*r*_(*t*) is the *N_q_* × *T_r_* analytic source signal to be estimated, ε_*r*_(*t*) is noise and *G* is the *N*_*s*_ × *N_q_* forward operator projecting source activity onto the sensors space. We emphasize here that although the ridge line is a non-linear filter, the ridge signal *w*^(*m*)^_*r*_(*t*) itself is linear with respect to data *M*(*t*) since the wavelet transform is a Iinear operation. The linear operator *G* is thus valid for ridge signals. In the present work, the estimation of the *N*_*q*_-dimensional *w*^(*q*)^_*r*_(*t*) is obtained through the Maximum Entropy on the Mean as developed (Amblard et al., 2004) and validated in (Grova et al., 2006). It is noteworthy that *w*^(*q*)^_*r*_(*t*) is an analytic source signal, which provides access to the true phase of the sources. All routines used for this article are coded in Matlab [The MathWorks Inc., Natick (MA), USA] is interfaced with Brainstorm and distributed as an open-access toolbox (http://neuroimage.usc.edu/brainstorm).

## Group-level synchronous networks

In order to perform group analyses, we first projected the time courses *w*^(*q*)^_*r*_(*t*) from the individual anatomy space onto the MNI brain template using routines implemented in Brainstorm (Tadel et al., 2011). On this common template, we characterized source activity inferred from the SEs under two different perspectives: (1) the power, proportional to the square of the amplitude of source activity during a SE, and (2) the connectivity, to infer functional networks emerging through phase synchrony. These two properties on the sources are complementary by definition, since phase synchrony and power are theoretically independent (but see Ghuman et al., 2010 for a link between source SNR and synchrony detectability). We note here that while power during SEs was computed at the source level, phase synchrony addressed connectivity within and among 88 parcels, each including around 200 sources (227 ± 136). For that purpose, we performed an initial clustering of cortical sources into 88 parcels derived from the Tzourio–Mazoyer anatomical atlas (Supplementary Figure 5). We computed both short-range and long-range connectivity based on these parcels. Short-range connectivity was computed as pairwise source connectivity within each parcel, whereas long-range connectivity was computed using local average signals within parcels.

### Power of synchronous sources

For each source *n* on the template, we quantified the source power underlying the SEs *r* detected for a subject *s* (hence the notation n,*r;s* in next Equation). First, we computed the mean energy *E*^(*q*)^:

(5) $$E_{n,r;s}^{\left(q\right)}=\frac{1}{T_{r}}\sum_{t\;=\;1}^{T_{r}}|w_{n,r;s}^{\left(q\right)}(t){|}^{2}$$

where *T_r_* is the number of time samples in the SE *r*. Given that wavelet coefficients *w*^(*q*)^_*n,r;s*_(*t*) are approximately 0-mean fluctuations, *E*^(*q*)^_*n,r;s*_ can be seen as a measure of source variance. We also compute the mean energy *E*^(*q*)^_*n,b;s*_ of the sources along ridges b located during a baseline period (−1.5 to −0.5 s before EEG spindle marker). The null hypothesis (H_0_) in our statistical test was that source variance has the same distribution during SEs than during baseline. We assessed this hypothesis using Fischer's test on a group statistic *F*. For each subject *s*, we ran 100 iterations where we selected a subset *R_i,s_* of 12 SEs, and a subset *B_i,s_* of 12 ridges in the baseline periods to compute the F-statistic as follows,

(6) $$F_{n,i,s}=\frac{\sum_{r\in R_{i,s}}E_{n,r}^{\left(q\right)}}{\sum_{r\in B_{i,s}}E_{n,r}^{\left(q\right)}},\;i=1,\ldots 100$$

Given that our subjects displayed at least 42 SEs (see Table 1), we could generate at least 2.9 × 10^5^ unique subsets *R_i,s_* and *B_i,s_* (21 SEs for each onset—late/early, 12 choices per combination). The average F-statistic over the 100 iterations, for each subject *F*_*n, s*_ was then computed. Finally, we averaged the statistics *F*_*n, s*_ over subjects in order to obtain the group-level average statistic *F_n_* We then derived the threshold *F*^*T*^_(12, 12)_ = 21.02 such that any sources *n* with *F*_*n*_ > *F*^*T*^_(12, 12)_ is significantly activated at a Bonferroni-corrected 5% level (*p* = 0.05/15028).

### Synchrony among sources

At this point, source signals *w*^(*q*)^_*r*_(*t*) are in a common anatomical space, thus we discard subject index. For each ridge signal *r* [we remind here that this signal is multivariate with dimensions (*Nsources* × *Nbins*)], we then computed pairwise synchrony ξ between parcels *i* and *j* using:

(7) $$\xi_{i,j}^{\left(r\right)}=\left|\frac{1}{T_{r}}\sum_{t=1}^{T_{r}}\frac{w_{r,i}^{\left(q\right)}(t)w_{r,j}^{\left(q\right)*}(t)}{\left|w_{r,i}^{\left(q\right)}(t)\right|\left|w_{r,j}^{\left(q\right)}(t)\right|}\right|$$

where *T_r_* is the length of ridge *r* and *w*^(*q*)*^_r,j_(*t*) denotes the complex conjugation of *w*^(*q*)^_*r,j*_(*t*). This definition of synchrony is equivalent to the phase-locking value (PLV, Lachaux et al., 1999) and provides added robustness to round-off error. For each pair (*i,j*), we thus computed *R* synchrony values, where *R* was the total number of ridges for a particular condition, then we averaged those values to obtain mean pairwise synchrony. For simplicity, we explain the synchrony computation and thresholding for a single pair of regions, but the same computations were performed for all pairs.

We assessed the statistical significance of synchrony strength using a non-parametric approach aimed at estimating the distribution of estimated synchrony under the Null Hypothesis, for each pair of parcels (*i,j*). To do this we used a shuffling approach by randomly permuting the identity of ridges, thus yielding:

(8) $$\xi_{i,j}^{\left(r,u\right)}=\left|\frac{1}{T}\sum_{t=1}^{T}\frac{w_{r,i}^{\left(q\right)}(t)w_{u,j}^{\left(q\right)*}(t)}{\left|w_{r,i}^{\left(q\right)}(t)\right|\left|w_{u,j}^{\left(q\right)}(t)\right|}\right|$$

where *r* ≠ *u* and *T* = min(*T_r_, T_u_*). By permuting all ridges for a particular condition, we constructed R shuffled values *on*ξ^(*r,u*)^_*i,j*_. We repeated this operation 100 times in order to ensure statistical robustness of our null hypothesis. The null hypothesis was that the distribution of phase-synchrony within a given ridge was equivalent to that generated from random combinations of the signals across ridges. The distribution of ξ^(*r*)^_*i,j*_ was then compared to the distribution under the null hypothesis and we derived a statistical threshold using the false discovery rate technique (see Supplementary Figure 2 for an illustration). This technique consists in finding the synchrony value ξ*^T^_i,j_* that ensures an arbitrary false positive rate (herein set to 5%). First, PLV scores ξ*_r,i,j_* were transformed to *z_r,i,j_* using Fischer's transform *z_r,i,j_* = 0.5 [ln (1 + ξ*_r,i,j_*) − ln (1 − ξ*_r,i,j_*)]. Then we computed the average z-scores *z_i,j_*, that were then inverse z-transformed to ξ*_i,j_* = exp (2*z_i,j_* − 1)/exp (2*z_i,j_* + 1). Finally, we consider regions pair (*i,j*) as being significantly synchronous if the average across SEs in each classes of ξ*_r,i,j_* is at least ξ*^T^_i,j_*. It is important to note here that the average PLV values and the PLV thresholds, derived respectively with equations (7) and (8), are computed specifically for each condition [(early, late) × (slow, fast)].

## Results

### MEG frequency-locking during spindles (SEs)

**Figure 3:**
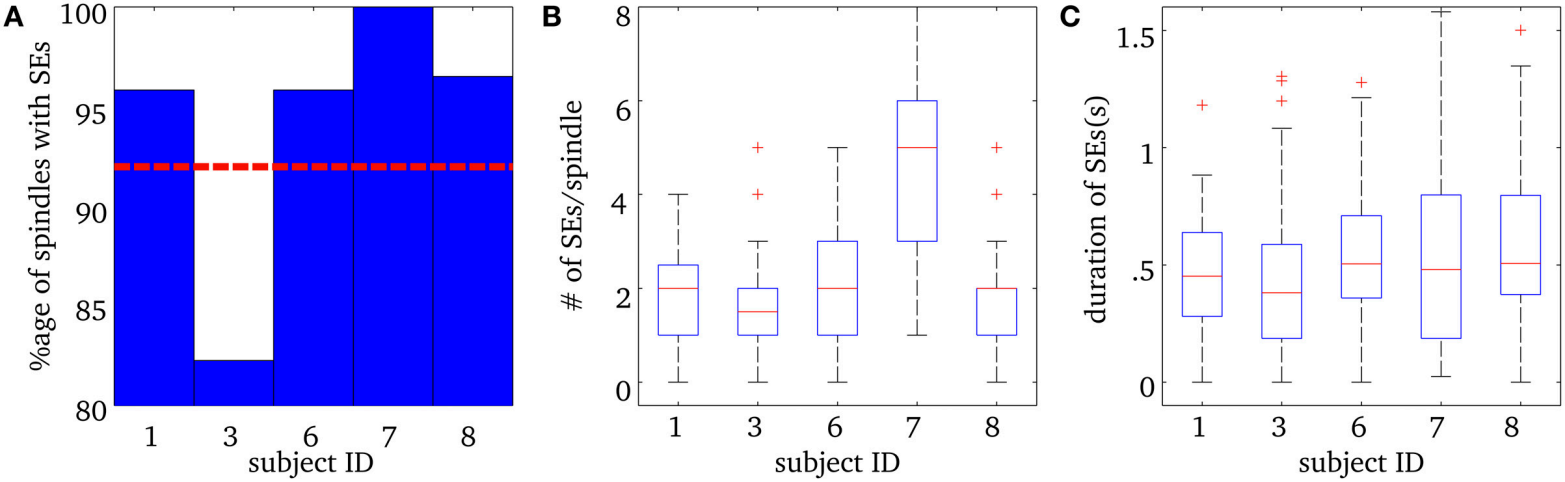
**Results for Synchrony Events (SE) in MEG sensor data during spindles. (A)** Percentage of spindles with at least one synchrony event (SE) for each subject. The horizontal red dashed line is the mean percentage over all subjects. **(B)** Number of SEs per spindle, for each subject. The box plots show the median number of SEs per ridge, in red, the 25th and 75th percentiles at the end of the box, and the “whiskers” indicate the minimum and maximum scores in the sample. The + in **(B)** indicate outliers. **(C)** Median total duration of SEs per spindle for each subject, along with the 25th and 75th percentiles. The + in **(C)** indicate outliers.

Figure 3 shows a number of descriptive statistics for the SEs observed at the MEG sensor level. More than 80% of EEG spindles for each subject had at least one significant MEG SE and the average was 92% (see Figure 3A). We note that frequency-locking was mostly sampled with 2 ridges per spindle for subjects 1, 3, 6, and 8 (Mean = 1.7 ± 1.1), while subject 7 had an average of about 5 ridges per spindle (Mean = 4.9 ± 3.0) (see Figure 3B). Ridges had a median duration of about 500 ms, which did not vary much across subjects, as shown in Figure 3C.

### Timing of MEG SEs during spindles

**Figure 4:**
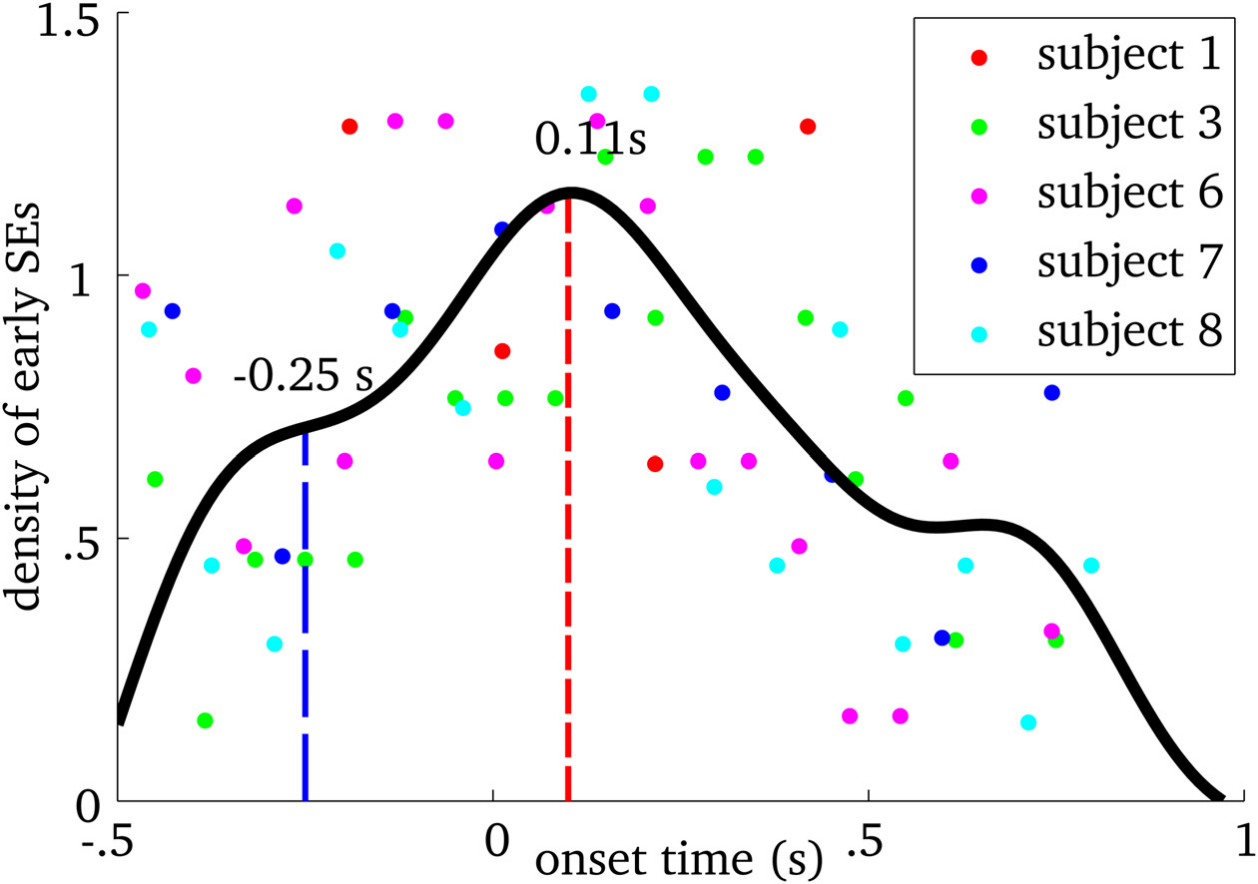
**Probability density plot of the onset time of the first synchrony event (SE) in MEG relative to the spindle onset time at Cz in the EEG, for each participant**. Each point on the graph shows the density of early SEs for a given onset time and subject. The black line is the spline interpolation of the empirical probability distribution. The blue dashed line indicates the first “plateau” and the red dashed line marks the mode of the distribution.

We examined when MEG ridges were first observed within spindles. Figure 4 shows the relative frequency of onset times. First SE from all spindles were pooled and using a probability density function, we computed their onset time with respect to EEG spindle marker at Cz. We observed that frequency-locking is initiated roughly between 250 ms before and 400 ms after EEG marker, with a main peak on the distribution at 110 ms after.

### Central frequency of SEs in spindles

**Figure 5:**
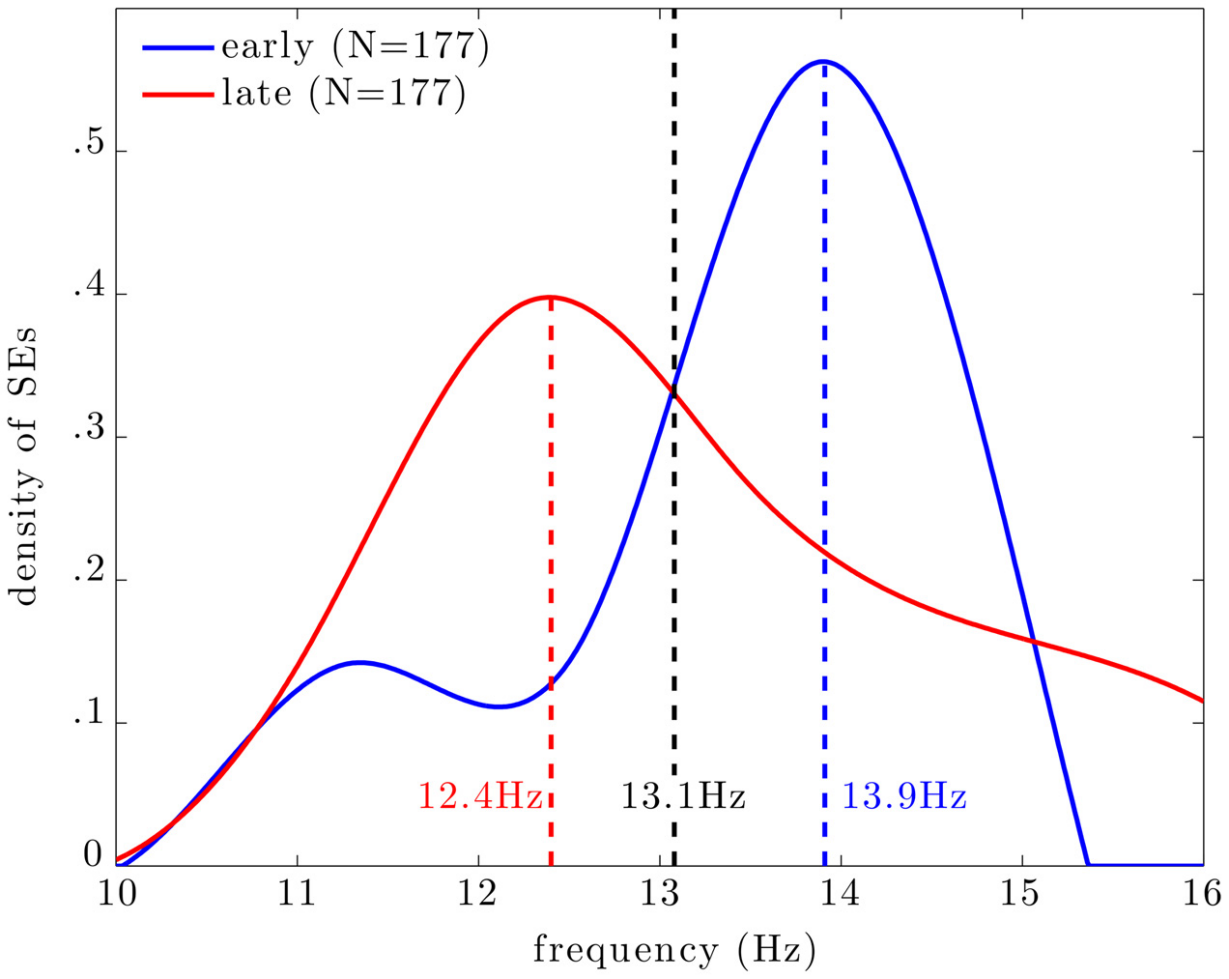
**Distribution of the central frequency of all SEs (dashed line), early SEs (blue line), and late SEs (red line)**. The distributions of frequencies of early and late SEs peak respectively at 13.9 and 12.4 Hz; these two distributions intersect at 13.1 Hz, which can be seen as an empirical frontier between slow (red) and fast (blue) SEs.

Figure 5 shows the distribution of central frequencies of all MEG SEs within EEG spindles (dashed line). The central frequency is here defined as the average instantaneous frequency along a SE. The distribution is bimodal with a main peak centered at 13.9 Hz and a lower peak around 11.5 Hz. Note that the spectral resolution of this analysis was limited to ~0.4 Hz due to the discrete and inhomogenous (i.e., with exponentially-spaced spectral bins) wavelet scaling. Taking into the spectral resolution of the analysis, we can state that the main frequency mode for MEG synchrony is between 13.4 and 14.3 Hz, and the lower mode is between 11.1 and 11.9 Hz.

Among all SEs, we select subsests of *early* and *late* events. Interestingly, the central frequency of early SEs, which are the first detected ridges relative to spindle onset, is mainly distributed around 14 Hz (blue curve). On the other hand, the central frequency of late SEs, which are the last detected ridge, is mainly distributed around 12 Hz (red curve).

### Activation maps

Supplementary Figure 3 illustrates cortical activations associated with SEs that take place either early, or late relative to spindle onset. These maps are displayed using Otsu's visualization threshold and allow a qualitative description of cortical activity linked to synchrony (Otsu, 1979). We can see that cortical energy is mainly distributed over the perirolandic cortex, bilaterally, for early synchrony. On the other hand, cortical energy is more broadly distributed for late synchrony and spans frontal, perirolandic, temporal, and occipital regions. It thus seems that cortical synchrony during spindles is initiated in fairly focal perirolandic regions and extends progressively to further regions.

**Figure 6:**
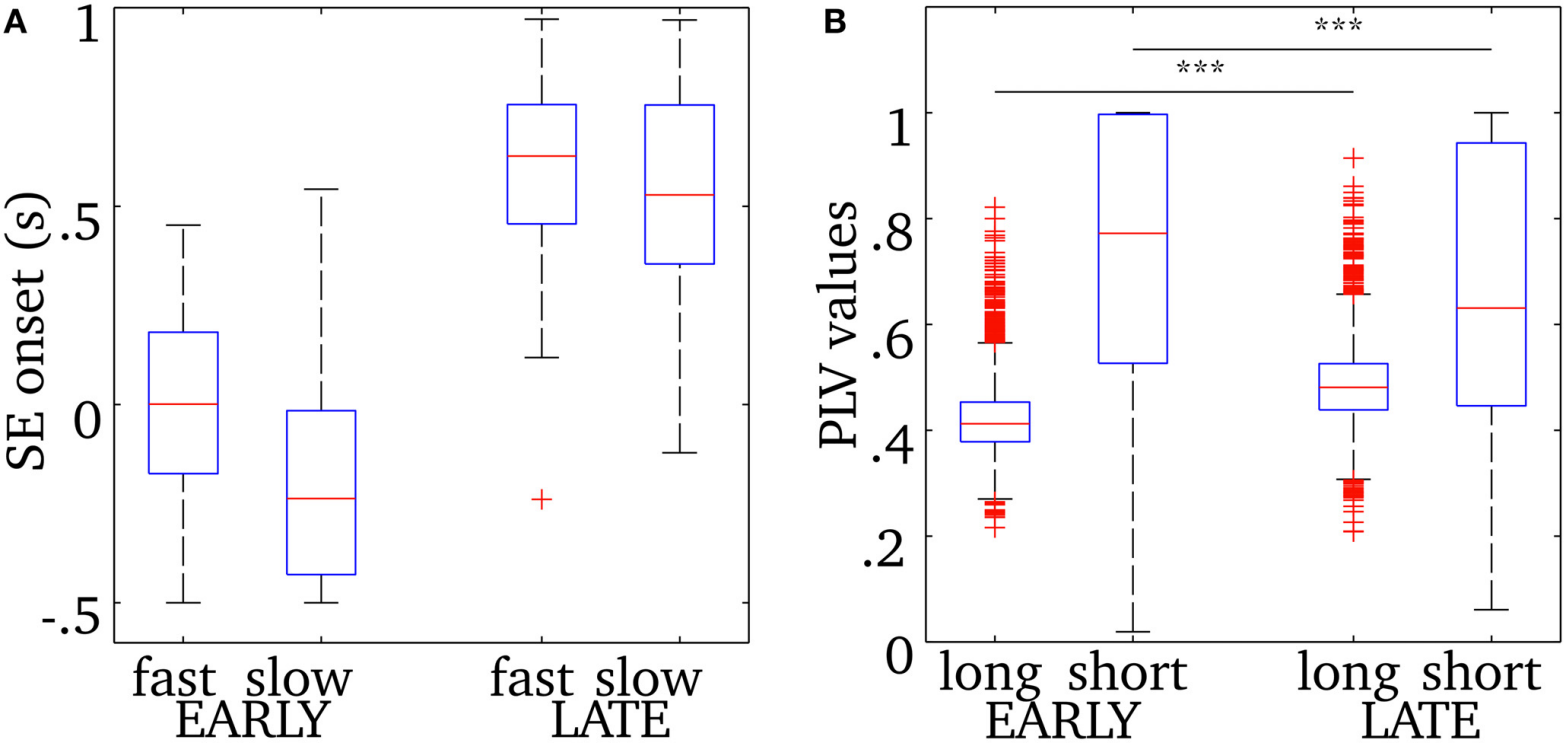
**(A)** Onset time descriptive statistics (median, quartiles, and extrema) for early or late events, with fast or slow oscillations, relative to the time of spindle onset defined on the EEG at Cz. **(B)** Phase-locking value descriptive statistics for long-range and short-range synchrony displayed for both early (columns 1, 2) and late (columns 3, 4) synchronous events. The horizontal bar and asterisks indicate a statistically-significant difference with *p* < 0.001 (see text for details). The number of points in the distributions of short-range PLV (source pairs) and long-range PLV (region pairs) are respectively 3,053,790 and 3828. See text for how events were classified.

**Figure 7:**
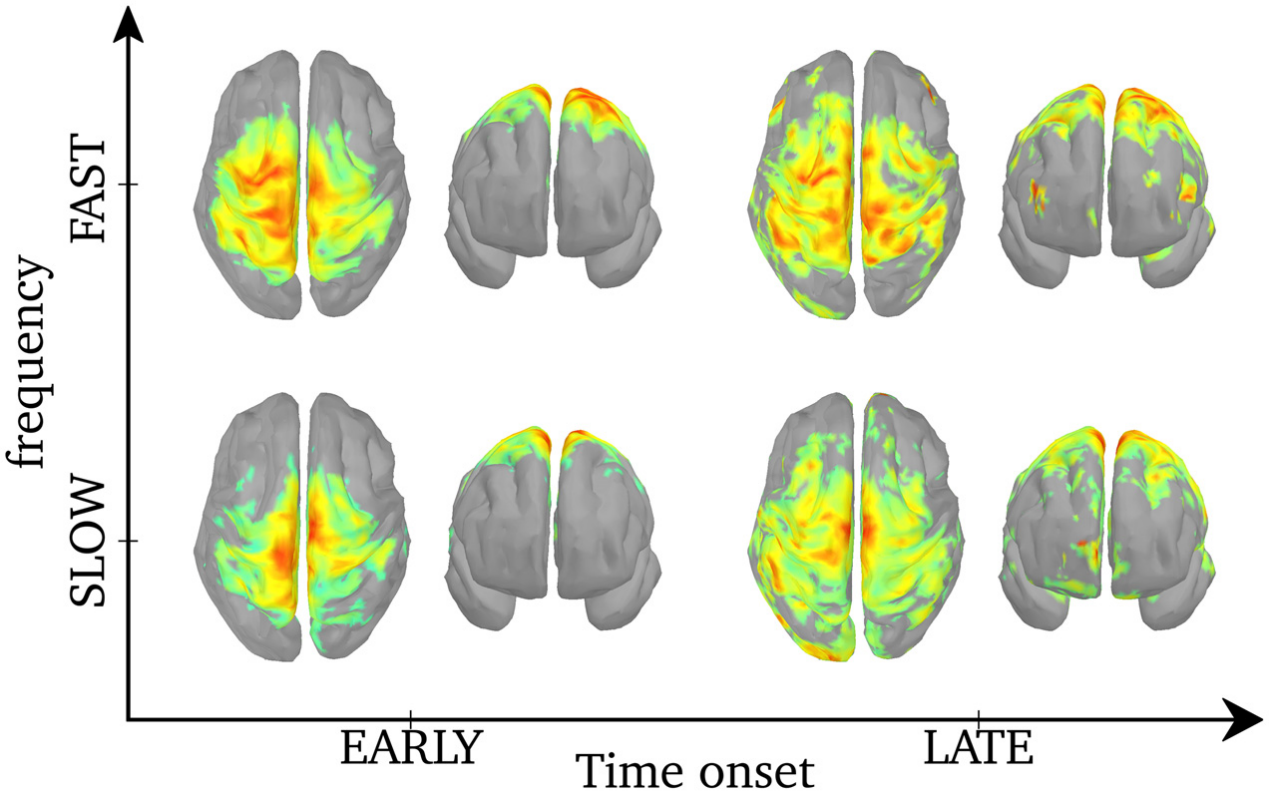
**Activation maps associated with each of the 4 categories of SEs**. The maps are normalized to a common scale (maximum power in red) and displayed using Otsu's threshold (Otsu, 1979). Based on the histogram of an object (vector or image), Otsu's threshold consists in classifying the object in two classes with minimal intra-class variances, then binarizing the object by setting the intensities of the lower class to 0 and that of the higher class to 1. Unthresholded activation maps are presented in Supplementary Figure 4.

As was shown in Figure 5, the central frequency of early SEs is mainly high but it can be low, and the reverse is true for late synchrony (mainly low, but can be high). Thus, the observed differences in cortical activation could either be due to the timing (early vs. late) or the frequency of synchrony (low vs. high) of synchrony. In order to disentangle the effects of these two factors, we pooled SEs with respect to each combination of timing and frequency. We first verify that, based only on the chronology of the synchronous events for each spindle, the distribution of the early and late events will sample unambiguously the early and late part of the spindles. This is shown in Figure 6. Using this approach, results in Figure 7 suggest that early SEs, no matter their frequency, emerge mainly from perirolandic regions. In addition, late synchrony emerges from a much broader set of regions, localized mainly in frontal, parietal, and occipital areas.

### Significant regions of cortical synchrony during sleep spindles

**Figure 8:**
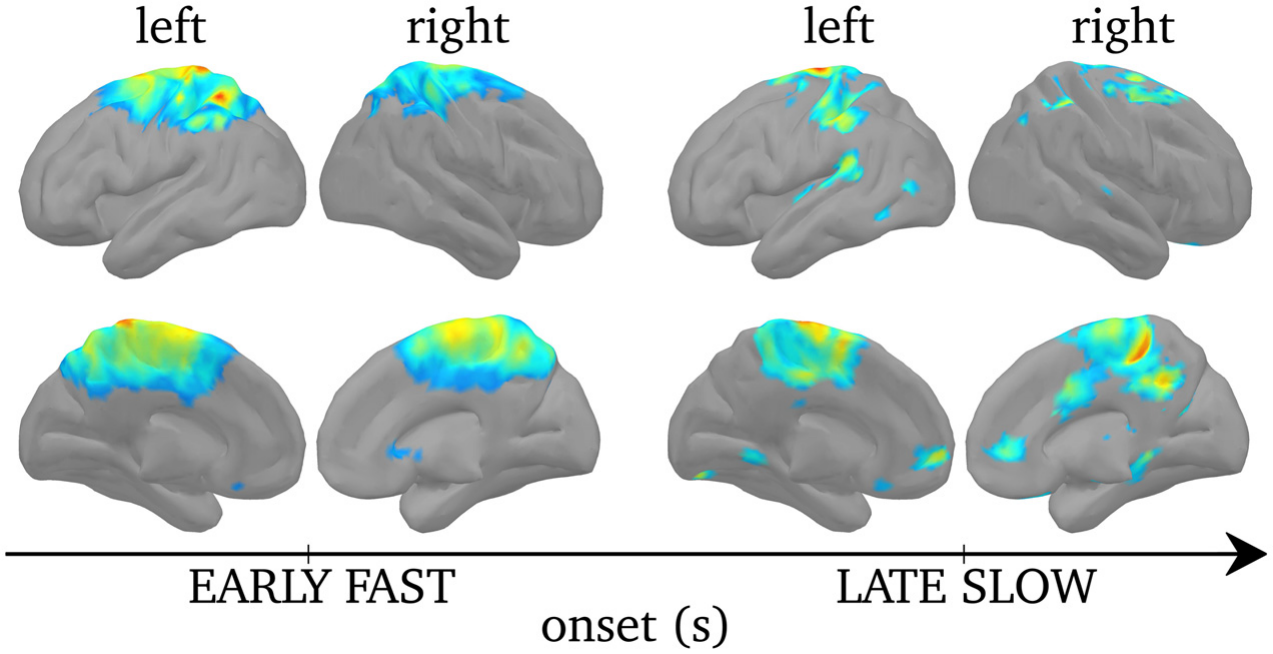
**Non-parametric statistical threshold on activation maps for the early fast SEs (left) and the late slow SEs (right)**. Upper rows displays cortices from a lateral view while lower row displays cortices from a medial view. Non-parametric statistical threshold was set to 0.05, Bonferroni-corrected. Color code here indicates the group-level average *F*-value (see Section Power of Synchronous Sources) of the significantly activated sources, insignificant ones are set to 0.

Figure 8 displays regions of significant projected power on cortical sources during SEs when the results were corrected for multiple comparisons using non-parametric statistical thresholding to Bonferroni-corrected *p* < 0.05. For early fast SEs, significant activations were found bilaterally, although stronger over the left hemisphere, in the postcentral gyrus, extending to the caudal part of the superior frontal gyrus, and in the left superior parietal lobule. In turn, for late slow SEs, activations were found, bilaterally, in the medial frontal gyrus, in the superior frontal gyrus, in the inferior parietal lobule and in the precuneus.

### Short- and long-range synchrony during sleep spindles

We examined separately short- and long-range synchronization during the early and late parts of spindles using measures of phase-locking value. Descriptive statistics for this analysis are displayed in Figure 6B. Overall short range synchronization, that is the averaged phase-locking values between pairs of sources within the same region, was significantly lower for late (0.63) than for early (0.77) synchrony [two-sample *t*-test, *t*_(3009)_ = 7.64, *p* < 0.0001]. On the other hand, long-range synchronization, that is the mean phase-locking value between all pairs of sources across distinct regions, was significantly higher for late (0.48) than for early (0.41) synchrony [two-sample *t*-test, *t*_(7654)_ = −38.87, *p* < 0.0001]. In particular, interhemispheric connections were denser in late synchrony, as the median PLV was increased by 0.085 in the latter condition [two-sample *t*-test, *t*_(3870)_ = 17.42, *p* < 0.0001, data not plotted]. Also, intrahemispheric increase of median long-range PLV value was much more marked in the right [Δ_PLV_ = 0.12, *t*_(1890)_ = 14.17, *p* < 0.0001, data not plotted] than in the left [Δ_PLV_ = 0.01, *t*_(1890)_ = 4.61, *p* < 0.0001, data not plotted] hemisphere.

### Synchronous networks during spindles

Recall from Section Group-Level Synchronous Network that we divided cortical regions into 88 distinct parcels. Phase-locking values (PLVs) were computed between all possible pairs of sources within each parcel to obtain short-scale synchrony values. In addition, we computed the average signal in parcel and computed PLVs between all possible pairs of parcels. Parcels were manually labeled to either the frontal, parietal, temporal, mesial or occipital regions. Supplementary Figure 6 shows a schematic representation of connectivity among and within cortical parcels, each being represented with a node. Long-range pairwise PLVs values greater than 0.8 are depicted, and links that are significant statistically are in bold. Statistical significance of the PLV value for a pair was determined using the approach described in Section Synchrony Among Sources. We computed, within each condition [(early, late) × (fast, slow)] the null distribution of large-scale synchrony in absence of SEs, i.e., using ridge signals from the baseline. From that distribution, we derive the FDR threshold above which synchrony is significant with p value of 5%. Short-range, within parcels synchrony, is coded with the node color and is not thresholded statistically.

Cortical networks involved a larger number of significant pairwise connections for late synchrony (99) than for early synchrony (31). In particular, interhemispheric connections were denser in late (8) than in early (1) synchrony (Supplementary Figure 6).

**Figure 9:**
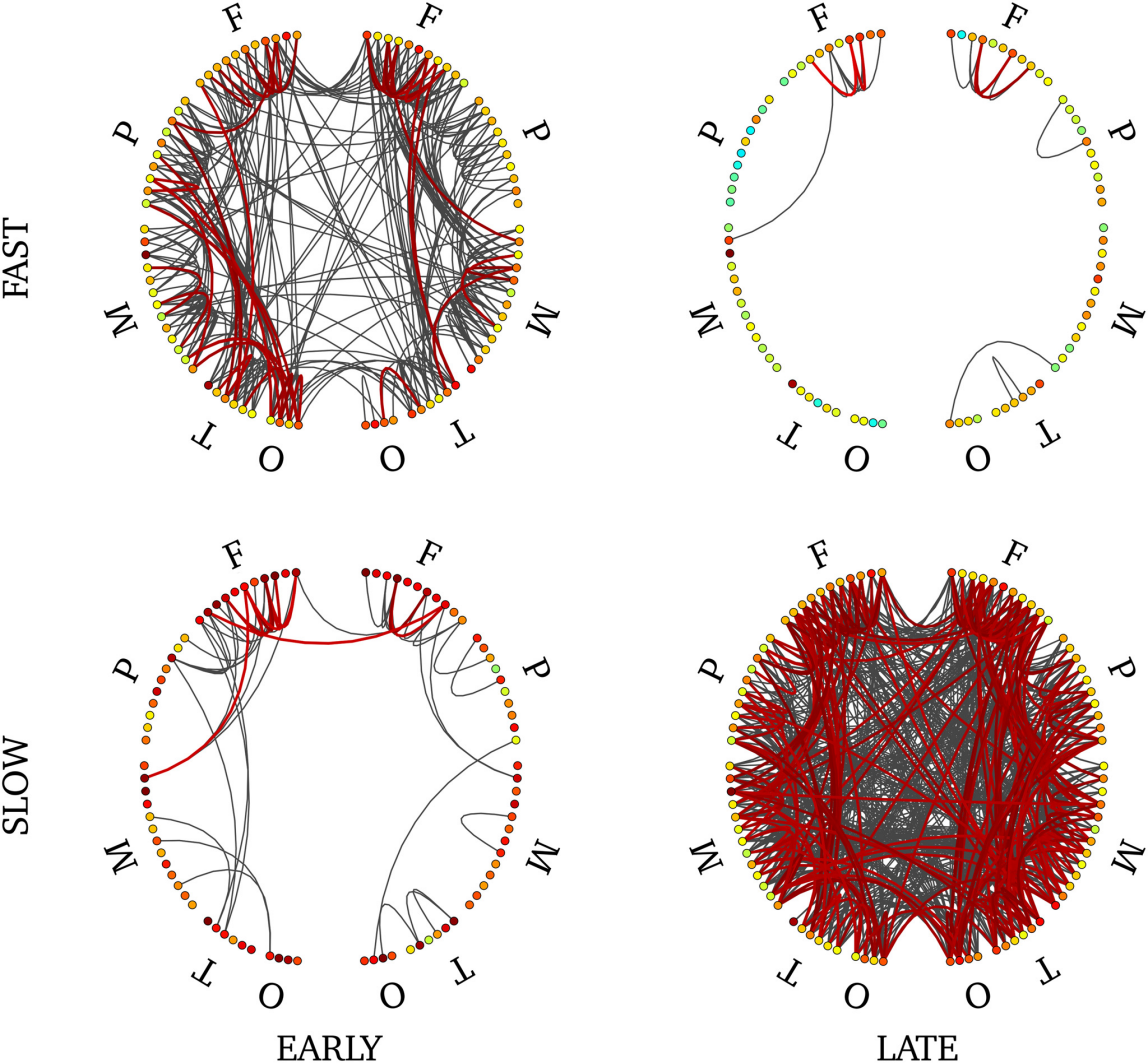
**Connectivity profile associated with early fast (upper left), early slow (bottom left), late fast (upper right) and late slow (bottom right) SEs**. Non-parametric FDR thresholding was applied to inter-regions PLV values and significant PLVs are displayed with thick lines. Visualization threshold was set to 0.8 and non-significant links are displayed with thin gray lines. Left and right halves of each plot reflect separate hemispheres, each consisting of 5 main divisions (F, frontal; P, parietal; M, mesial; T, temporal; O, occipital). Node colors reflect intra-region synchrony (no threshold applied).

In order to disentangle effects of timing versus frequency, we analyzed separately the 4 combinations of these two factors. We show the statistically-significant PLV links in Figure 9 for late slow and early fast synchrony where we observed significant pairwise connections. There were no significant connections in the other two conditions (early slow, late fast). Interstingly, late slow synchrony involved a larger number of connections (137) than early fast synchrony (31). Finally, significant interhemispheric synchrony was observed only in late slow synchrony. As a confirmatory analysis, we verified that this pattern was also observable on individual subjects' connectivity profiles (see Supplementary Figure 7). We found this effect was observable on 4 out 5 subjects, whereas the last subject showed an overall low number of interhemispheric links.

## Discussion

In this work, we addressed the dynamics of neuronal networks during sleep spindles under the angle of phase synchrony. We proposed an original source imaging approach to reveal the cortico-cortical functional connectivity associated with transient synchronous events occurring during sleep spindles. We discuss the present work in two steps: (1) the validation of the proposed ridge-based methodology against consensual knowledge on spindles and (2) the interpretation of new findings in relation to hypothesized functional roles of spindles.

### Validation of ridges for the study of spindles

The following sections are intended to validate the use of frequency-locking for characterizing the dynamics of cortical activity during sleep spindles. We argue and provide supporting evidence that frequency-locking during spindles reveals spectral and topographical properties that were previously reported by studies on the signal amplitude during spindles. In addition, we show that imaging the power of cortical sources underlying frequency-locking during spindles yields activations within regions that were previously shown to be involved in spindles using a variety of imaging techniques. The results discussed in this first section will allow us to argue that amplitude-based and synchrony-based features of spindles reflect similar neurophysiological processes.

#### Detectability of frequency-locking spindles

We used a wavelet-ridge framework to detect and quantify frequency-locking during spindles. Using this framework, we observe significant MEG SEs in the vast majority of spindles and subjects, and the method allowed us to measure the duration of spindle-related frequency-locked activity with remarkable consistency across subjects. We see two main reasons why wavelet ridges should be favored for studying frequency-locking during spindles. (1) We observed that the central frequency of SEs detected on MEG sensors is higher earlier compared to later within spindles. (2) It was shown that cortical sources vary during the time course of spindles recorded in MEG (Dehghani et al., 2011), which is consistent with the observation that spindles are observed different MEG sensors along time (Hao et al., 1992; Zierewicz et al., 1999). Frequency-locking recorded with MEG thus reflects a non-stationary process.

Therefore, global measures computed over the entire duration of spindles, such as magnitude-squared coherence, cannot capture the complexity of the dynamics underlying synchrony during spindles, which may explain why they yield low (0.22) synchrony values (Dehghani et al., 2010; Bonjean et al., 2012). Another approach based on autoregressive modeling and partial cross-coherence also yielded low values (−0.29 to 0.38) for average MEG synchrony (Langheim et al., 2006). However, instead of capturing the complexity of MEG synchrony, this latter approaches filters out non-stationary components of MEG signals and estimates coherence on the residue. In contrast, wavelet ridges are particularly well suited to reveal patterns of frequency-locking that change over time and space, because their detection is more robust to spectral or spatial perturbations (Amor et al., 2005).

#### MEG spindle dynamics

Our results showed that frequency-locking has a higher frequency when it appears at the beginning of a spindle and lower frequency when it appears at the end, with a clear boundary at 13 Hz. This corroborates previous studies reporting that intra-spindle frequency is frequently characterized by a progressive slowing of oscillatory activity (Schonwald et al., 2011). We also observed a typical 500 ms delay between early and late synchrony. Using automatic spindles detection based on signal energy, Dehghani et al. (2011) showed that spindles in MEG could arise up to 200 ms before their EEG counterpart. Interestingly, from the perspective of synchrony, a similar delay can be observed between the onset of spindles visible on the EEG and MEG synchrony (MEG often earlier). On average, however, MEG synchrony arises 110 ms after EEG spindles onset.

By localizing the ridge complex signal, we efficiently target the sources that generate frequency-locking during MEG spindles. The ridge signal is thus more appropriate for the study of functional connectivity, as will be discussed in the next section. From the perspective of average power, we find different cortical activation maps for ridges with higher versus lower central frequency. Earlier and faster SEs emerged mainly from centro-parietal regions bilaterally, but only the postcentral gyrus and the superior parietal lobule survived statistical thresholding. Other groups also linked fast spindles to centro-parietal sources using dipolar source modeling (Manshanden et al., 2002; Urakami, 2008), distributed source modeling (Anderer et al., 2001), spatial filtering (Gumenyuk et al., 2009), and fMRI (Schabus et al., 2007). On the other hand, later and slower SEs emerged, bilaterally, from frontal (medial and superior gyri), and parietal (precuneus, inferior parietal lobule). Activation of the medial frontal lobe for slow spindles was also observed using distributed source modeling (Anderer et al., 2001) and fMRI (Schabus et al., 2007). We note here that despite the small sample size in our study (5 subjects), our source localization yields highly significant activity with remarkable concordance with the literature.

In addition, it was reported that frontal activity linked to slow spindles shows fair inter-subject variability both at the sensors (Doran, 2003) and the sources level (Anderer et al., 2001), thus group analyses would tend to dampen activity in this region. Inter-subject variability could also be explained by lower Signal to Noise Ratio (SNR) for signals generated by deep/mesial sources, which impacts on the performance of any sources localizer (Hämäläinen and Ilmoniemi, 1994). The significant group activation in medial frontal gyrus could thus be explained by higher resistance of ridge-based source localization to lower SNR (Zerouali et al., 2013).

### New insights from functional connectivity

#### Sources of synchrony: connectivity

As discussed in Section Group-Level Synchronous Networks, short-range connectivity is assessed using pair-wise synchrony within parcels (3,053,790 pairs in total) while long-range connectivity was defined as pair-wise synchrony among regions (3828 pairs). We observe that short-range spindle synchrony (99.9% of all cortical pairwise associations) was significantly higher for earlier than for later SEs, while the reverse was true for long-range synchrony (higher for later SEs). This observation supports the view that short- and long-range synchronies are somewhat antagonistic. Indeed, short-range synchrony must be weak for a network to synchronize massively among long-range distances (Langheim et al., 2006) and strong short-range synchrony, such as during slow wave sleep, prevents TMS-induced electrical waves from propagating and reaching far cortical targets (Massimini et al., 2005). We however note here that our values of short-range synchrony are corrupted by current leakage during source reconstruction. Indeed, due to the ill-posed nature of the sources imaging inverse problem, source extension is usually overestimated, thus creating artificially high PLV values (Schoffelen and Gross, 2009; Hillebrand et al., 2012).

Our most important result is that, regardless of the timing of frequency-locking (early vs. late SEs), we observed strong fronto-temporal connectivity, bilaterally. However, inter-hemispheric connectivity was weak during early SEs but was significantly strengthened during later SEs. Also, although highly significant, the quantitative variations in long-range functional connectivity are weak (ΔPLV = 0.03). In our work, a 6% (ΔPLV/PLV_early_) increase in global synchronization level of the cortex yielded a 200% [(99 − 31)/31] increase in the number of significant long-range connections. This is an interesting observation since it supports the view that the reinforcement of long-range connections of the functional networks during spindles is a low-cost mechanism. Cost-efficiency is an important feature of small-world networks, such as brain networks, which optimize the balance between local and long-range connectivity in order to minimize wiring cost while preserving efficient information flow (Bassett and Bullmore, 2006). It is worth to mention that the null-hypothesis models the synchrony among uncoupled oscillators with similar frequency contents (due to the narrow-band spectrum as displayed in Figure 5). It has been computed by shuffling the time series in sources space, separately in each condition. Alternatively, we could have modeled the null hypothesis as asynchronous events at the sensors level. This could have been done by shuffling the ridge masks among spindles in the data space. On a qualitative basis, we observe that both approaches yield equivalent thresholds, thus similar connectivity graphs. In addition, it would be of interest to compare the connectivity changes highlighted by our statistical thresholding of connectivity matrices to other dimension-reduction strategies, such as minimum spanning trees (Tewarie et al., 2014).

Taken together, our results suggest that functional connectivity undergoes important changes during spindles, evolving from a pattern of short-range and intra-hemispheric connections to more long-range and inter-hemispheric connections. This transition from local to global networks during spindles is one of the most important new discoveries from our work.

#### Sources of synchrony: dynamics

Most spindles started with a faster oscillation that decelerated to a slower oscillation at the end of the spindle. This suggests that fast and slow stages of spindles are two manifestations of the same oscillator, which we view as a neural system endowed with functional capabilities, that varies in frequency over a dynamic range. The fast/slow spindle classification thus may result solely from the relative durations of the fast and slow regimes.

One puzzling observation is that early SEs can be either fast or, although infrequently, slow and the reverse is true for late SEs. We thus asked what is the fundamental property underlying the two classes of spindles, timing or frequency? We found that, for both early and late synchrony, cortical power has a consistent distribution regardless of frequency. On the other hand, functional connectivity patterns are inconsistent with respect to either timing or frequency alone, early slow and late fast synchrony being much reduced compared to the early fast and late slow synchrony.

It is noteworthy that we observe a link between the frequency at which the functional network oscillates and its spatial extent. Indeed, we showed that early SEs, which are characterized by a high frequency (>13 Hz), involve lower large-scale connectivity than late SEs, which are characterized by a lower frequency (<13 Hz). Despite a small frequency range, this result is consistent with evidence suggesting that fast rhythms (i.e., gamma) support local synchrony among neurons within a cortical patch while slower rhythms (i.e., beta, alpha, theta) support distant synchrony (von Stein and Sarnthein, 2000). The coupling mechanism between frequency and spatial extent was shown to rely on the firing properties of interneurons in a mathematical model of coupled networks. Indeed, a qualitative change in interneuron firing (spike doublet) was shown to cause a switch in oscillating frequency from gamma to beta range (Ermentrout and Kopell, 1998). Interestingly, using similar model, it was shown that quantitative changes in the level of self-inhibition of interneurons could tune the oscillating frequency within the lower beta range (12–20 Hz, Kopell et al., 2000). Accordingly, we can hypothesize that, during the time course of a spindle, the levels of self-inhibition of interneurons of the thalamo-cortical network increase, thus causing the oscillation frequency to slow down.

In the light of previous findings, our results show that, although frequency does not impact on the sources involved in synchrony, the connectivity of the network is certainly dependent on appropriate time-frequency dynamics that might be modulated through self-inhibitory properties of interneurons.

#### Implications for studies on the functional role of spindles

The implication of spindles in the consolidation of memory has been suggested by a wealth of studies and is now widely accepted as unequivocal (Walker and Stickgold, 2006). Procedural learning and declarative memory are associated to spindle density and sigma power (Morin et al., 2008; Schabus et al., 2007; Tamaki et al., 2009; Barakat et al., 2011; Fogel et al., 2012). Generators of the oscillatory regime and functional connectivity underlying early and late synchrony may underlie the role of spindles in brain plasticity. Future research should investigate how overnight procedural and declarative memory consolidation would influence generators and functional connectivity of early and late spindle synchrony. This research should also be performed in an older population, which not only shows reduced spindle density, but also reduced spindle amplitude, duration, and a trend for faster spindle mean frequency. Age-related difference in overnight memory consolidation (Spencer et al., 2007; Aly and Moscovitch, 2010; Wilson et al., 2012) may be linked to modifications in functional connectivity of spindle synchrony.

## Conclusion

In this paper, we studied sleep spindles as a sequence of transient synchronous events using MEG recordings. The methodology we developed targets specifically cortical synchronous oscillations. It involves a non-linear filtering of MEG signals using wavelet ridges, yielding ridge signals on the sensors that embed the synchronous component buried in MEG recordings. Our approach is endowed with a high sensitivity to spindle activity, since synchrony can be detected regardless of energy, and high specificity due to a controlled selection of synchronous events. We were thus able to extract statistically robust patterns of functional connectivity despite having tested only five participants. We were able to show that functional connectivity undergoes dynamical changes with respect to time-frequency features of the spindles. Future research will focus on the effect of aging and learning on such functional connectivity.

### Conflict of interest statement

The authors declare that the research was conducted in the absence of any commercial or financial relationships that could be construed as a potential conflict of interest.

## Supplementary material

Supplementary Figure 1 **Superimposition of the real part of the ridge signal with the original signal of the Figure 1**.

Supplementary Figure 2 **Example of FDR statistical thresholding of PLVs distribution**. The blue and red curves represent the distributions of PLVS during a SE and during a baseline period, respectively. The threshold (vertical dashed line) is set such that the ratio between the suprathreshold area under the red curve and the suprathreshold area under the blue curve is equal to an arbitrary value. We chose to set the FDR threshold at 5%, which amounts to tolerate 5% false positives.

Supplementary Figure 3 **Activation maps associated with early (upper left) and late (upper right) SEs**. he maps are displayed with Otsu's threshold for easier visual comparison.

Supplementary Figure 4 **Unthresholded activation maps associated with each of the 4 categories of SEs**. Normalization and color code are the same as Figure 7.

Supplementary Figure 5 **Cortical parcels used in the computation of large-scale functional connectivity**. Parcels are grossly derived from the Tzourio-Mazoyer atlas and registered with the MNI template. Color-coding indicates brain lobes: frontal (red), parietal (blue), temporal (cyan), medial (green), and occipital (orange).

Supplementary Figure 6 **Connectivity profile associated with early (left) and late (right) SEs**. Inter-region synchrony is depicted with curved lines linking two nodes. Color coding and statistical thresholds are the same as in Figure 9.

Supplementary Figure 7 **Connectivity profiles associated with early (upper row) and late (bottom row) SEs for each subject**. No statistical threshold was computed on these profiles since subject-based analysis suffers low degrees of freedom; all links displayed reflect PLV values overs 0.9.

Supplementary Figure 8 **Morse wavelet parameterized with β = 4 and γ = 4. (A)** Wavelet representation in the time domain. The thin black and dashed lines represent respectively the real and imaginary parts of the complex wavelet and the thick line represents its envelope. **(B)** Representation of the wavelet in the Fourier domain over the positive part of its spectrum.
