# Supplementary material for: A time-frequency analysis of the dynamics of cortical networks of sleep spindles from MEG-EEG recordings
Source: Front Neurosci. 2014 Oct 28;8:310. doi: 10.3389/fnins.2014.00310 (PMC4211563; doi:10.3389/fnins.2014.00310)
Supplement: Supplementary file 1 [file Presentation1.PDF]

1. Appendices

1.1 Morse wavelets

The Morse family of wavelets is time-frequency atoms defined analytically in the Fourier domain by the expression (Lilly & Olhede, 2012):

$$\psi_{\beta,\gamma}(\omega) = U(\omega)\,\omega^{\beta}\,e^{-\omega^{\gamma}} \quad \text{(A.1)}$$

where ω is the frequency and $U(\omega)$ is the Heaviside step function. This function of frequencies can be seen as a filter, the shape of which is determined by parameters β and γ. β is the number of vanishing moments of the wavelet, i.e. the polynomial drift order up to which the wavelet coefficients cancel. γ characterizes the decay of the filter at high frequencies and controls the spectral resolution of the filter. It is noteworthy that the time support of the wavelet is linked to both parameters; the number of significant cycles within that support is given by $\sqrt{\beta\gamma}/\pi$. The wavelet parameters were set to $\beta = \gamma = 4$ in this study, so that the wavelet one main oscillation. The shape of the filter and the temporal expression of the wavelet are shown in supplementary figure 8. This choice of parameters makes the filter symmetric (see supplementary figure 8b), which is important for ridge estimation (see Lilly & Olhede, 2010 for details).

Morse wavelets have the interesting property of being analytic as their coefficients are null on the negative part of the spectrum. The wavelet coefficients on the time-frequency plane, which is computed as described in section 2.2, encode the instantaneous amplitude and phase of the analyzed signal that can be recovered approximately using wavelet ridges, as discussed in section 2.3.

1.2 Wavelet ridge analysis

Let's consider a wavelet $\Psi(t)$, with central frequency η, that is scaled by a factor a and translated in time by a factor b. A dictionary of wavelet functions can be constructed by dilating and translating the wavelet over an infinite range of parameters, which can be written in the time domain:

$$\Psi_{(a,b)}(t) = \frac{1}{\sqrt{a}}\,\Psi\left(\frac{t-b}{a}\right) \quad \text{(A.2)}$$

and in the Fourier domain (the hat notation denotes the Fourier transform):

$$\widehat{\Psi}_{(a,b)}(\omega) = \sqrt{a}\,\widehat{\Psi}(a\omega)\,e^{-i2\pi\omega b} \quad \text{(A.3)}$$

The central frequency of the dilated wavelet is given by $\xi = \eta/a$. Let's consider now an analytic signal of the form $s_{+}(t) = A_0(t)e^{i\phi(t)}$. The wavelet transform of this signal is performed by its projection on the dictionary of atoms, yielding time-frequency coefficients

$$T(a,b) = \sqrt{a}\int_{-\infty}^{+\infty} \hat{s}_+(\omega)\overline{\widehat{\Psi}(a\omega)}e^{i2\pi\omega b}\,d\omega \quad \text{(A.4)}$$

that reflect the local oscillatory behavior of the signal around time b and frequency ξ. As discussed in section 9.1, the wavelets used in this work are the Morse wavelets defined by (A.1) (Lilly & Olhede, 2012). By inserting the Morse wavelet into equation (A.4), we obtain the normalized scalogram $S(a,b)$ defined as follows:

$$S(a,b) = \frac{|T(a,b)|^2}{a} = \frac{1}{4}\,A_0^2\,\eta^{2\beta}\,(2-\frac{\phi'(b)}{\xi})^{2\beta}\,e^{-2\eta^{\gamma}(2-\frac{\phi'(b)}{\xi})^{\gamma}} \quad \text{(A.5)}$$

Equation (A.5) is fundamental because it shows that, for any given time b, the maximal amplitude of the scalogram is found when the instantaneous frequency of the signal, i.e $\phi'(b)$, equals the central frequency (ξ) of the dilated Morse wavelet. In practice, this means that we can estimate the instantaneous frequencies of a signal by locating the local maxima of $S(a,b)$ defined by:

$$\frac{d}{da}|T(a,b)|^2 = 0$$

$$\frac{d^2}{da^2}|T(a,b)|^2 < 0 \quad \text{(A.6)}$$

It is noteworthy that solution to equation (A.6) might not be unique, as one can have many local maxima. Amor et al. (2005) suggested to filter out local maxima with energy less than 95% of that of the global maximum. Then, when local maxima are contiguous in the time-frequency plane, they form a *ridge* that indicates the occurrence of a persistent oscillatory mode in the data (as illustrated in figure 1a).

1.3 Maximum Entropy on the Mean (MEM)

Distributed source imaging models the recorded data $m(t)$ as a linear superposition of source intensities $\mathbf{q}(t)$, mixed by the forward operator G:

$$m(t) = G\mathbf{q}(t) + \varepsilon \quad \text{(A.8)}$$

where ε is measurement and physiological noise. The operator G is of dimensions [$N_{sensors}$ x $N_{sources}$] and each column of that matrix represents the magnetic field produced by unitary activity of one source to all sensor. As mentioned in section 2.1, *G* is obtained as the analytical solution to Maxwell's equations for sources in a spherical head model. The inversion of model (A.8) is performed using the MEM framework in order to estimate the source intensities.

The MEM is a non-linear Bayesian approach to solve an inverse problem using metrics derived from information theory. As for any Bayesian framework, MEM relies on the definition of a prior model of source activity, which is updated using the data to produce an estimate of source activity. The main difference between the MEM and usual Bayesian solution is the informational based metric used to update the prior model. This metric is the μ-entropy that defines a distance between a probability density law $p(\mathbf{q})d\mathbf{q}$ and a prior model $\mu(\mathbf{q})d\mathbf{q}$:

$$S_\mu(p) = -\int p(\mathbf{q}) \log\frac{p(\mathbf{q})}{\mu(\mathbf{q})}\, d\mathbf{q} \tag{A.9}$$

As shown by (A.9), the μ-entropy is maximal (equal to 0) when $p(\mathbf{q}) = \mu(\mathbf{q})$ (in absence of data, the best solution is the prior) and negative otherwise. By maximizing the μ-entropy, the MEM finds the closest solution to model, i.e. the one that updates it in order to explain the data. This framework makes minimal assumptions about missing information from the data (Jaynes, 1957) and offers the best solution to inverse electromagnetic problems (Rice, 1990). The definition of a physiologically realistic prior source model $\mu(\mathbf{q})d\mathbf{q}$ is crucial to estimate accurately the source intensities. The following section exposes the prior model used in this work.

Prior source model

The prior model consists in describing the brain functional activity using K homogenous cortical parcels P_k. Each parcel gathers n_k dipoles and is characterized with a hidden binary state variable S_k, (1=active state; 0 = inactive state). We denote $\alpha_k = p(S_k = 1)$. The prior probability distribution $\mu(\mathbf{q})d\mathbf{q}$ of the sources within parcel k is defined as a mixture between both states :

$$\mu(\mathbf{q})d\mathbf{q} = [(1-\alpha_k)\delta(\mathbf{q}_k) + \alpha_k N(\nu_k, \Sigma_k)\mathbf{q}_k]\, d\mathbf{q}_k \tag{A.10}$$

where $\delta(\mathbf{q}_k)$ is the Dirac distribution modeling the inactive state and $N(\nu_k, \Sigma_k)$ is a n_k-dimensional multivariate normal distribution of mean ν_k and covariance Σ_k modeling the active state of parcel k. As we assume that the cortical patches are independent, the prior model $d\mu(\mathbf{q})$ is written as

$$d\mu(\mathbf{q}) = \prod_{k=1}^{K} d\mu_k(\mathbf{q}_k) \tag{A.11}$$

The probabilities α_k are computed using the multivariate pre-localization formalism (MSP, Mattout et al., 2004). The MSP scores, ranging between 0 and 1, measure the ability of each source to explain the data. The parcellization of the cortical surface is obtained from a region growing algorithm on the sources sorted according to decreasing MSP scores. In the present work, the size of the parcels is limited to a neighborhood order 3, thus each parcel typically gathers 30 dipoles. We then define:

$$\alpha_k = median\ (s_k) \tag{A.12}$$

where s_k is the MSP scores of the n_k sources within the parcel k. The probabilities α_k is subordinate to the definition of the parcels.

MEM performance

The MEM solution was shown to be among the most accurate methods for localizing sources of both EEG (Grova, 2006) and MEG signals (Chowdhury, 2013). In particular, it is able to delimit the spatial extent of the cortical generators, which can be interpreted as a good specificity. Recently, Chowdhury et al. (2014) showed that the MEG/EEG fusion in the MEM framework achieves even higher localization accuracy. This avenue is a promising approach for refining the analysis of functional connectivity.
