# Supplementary material for: A time-frequency analysis of the dynamics of cortical networks of sleep spindles from MEG-EEG recordings
Source: Front Neurosci. 2014 Oct 28;8:310. doi: 10.3389/fnins.2014.00310 (PMC4211563; doi:10.3389/fnins.2014.00310)

Supplementary Figure 1

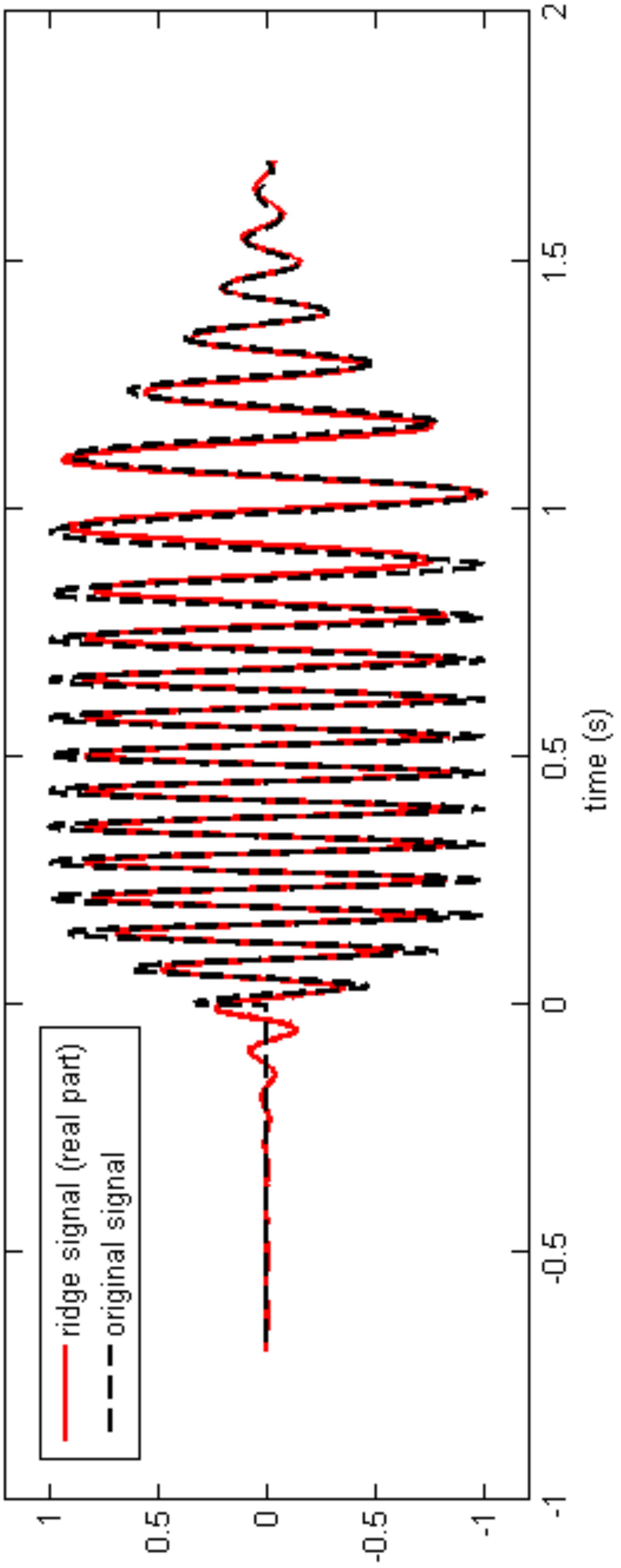

Supplementary Figure 2

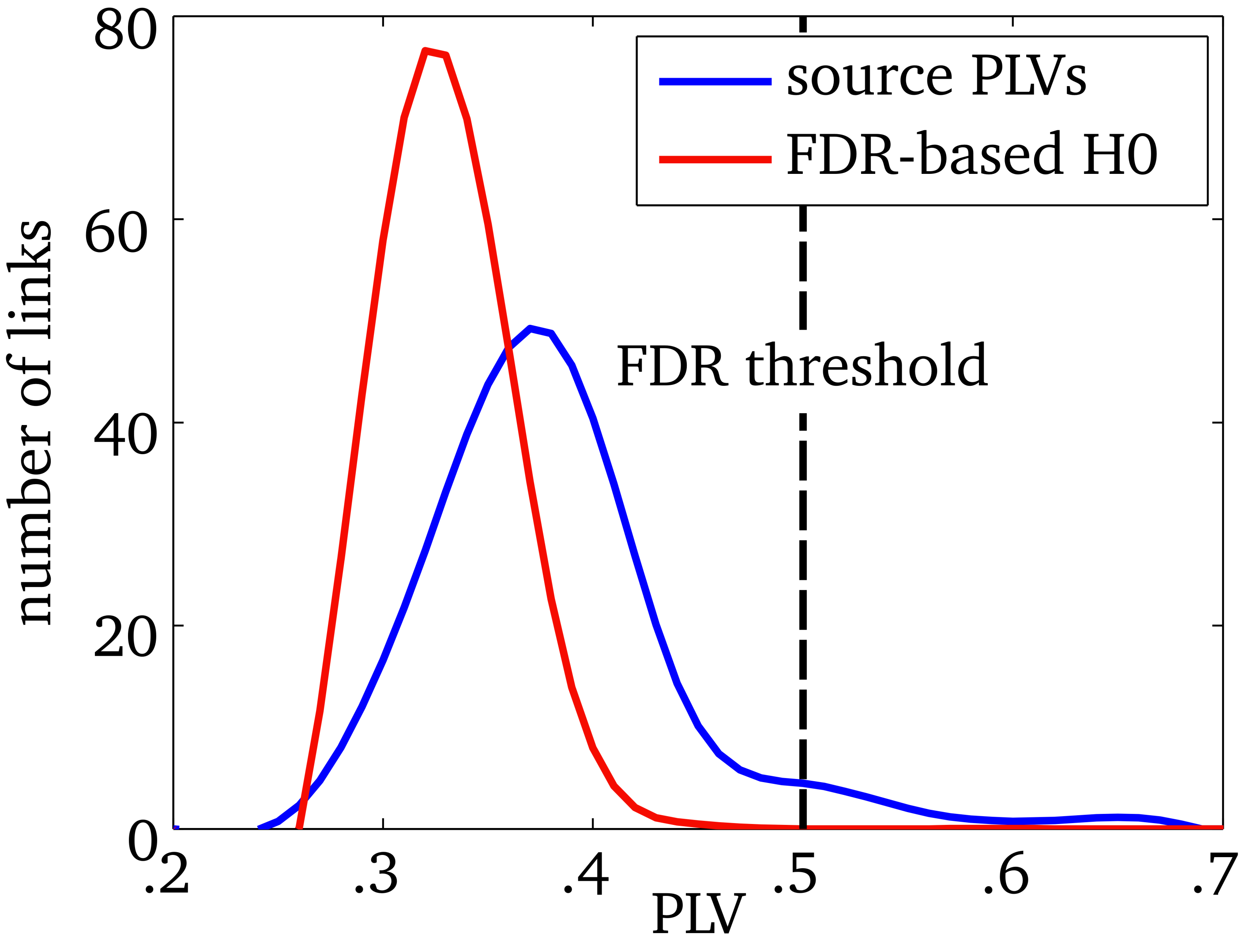

Supplementary Figure 3

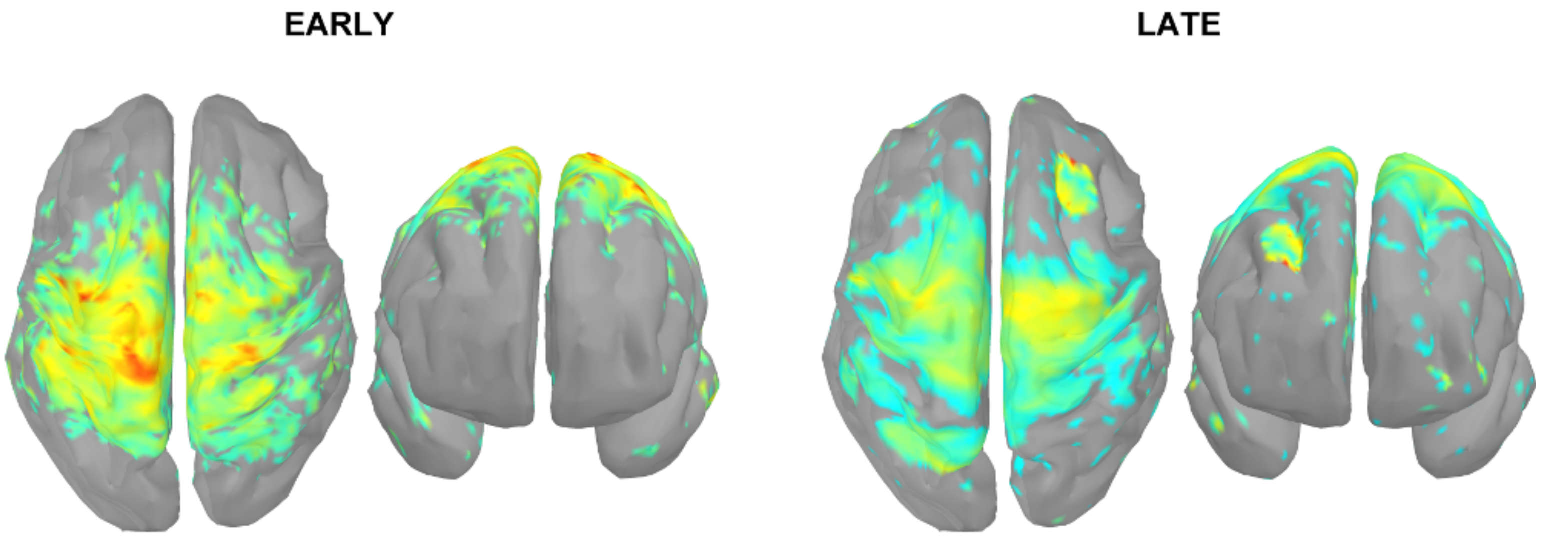

Supplementary Figure 4

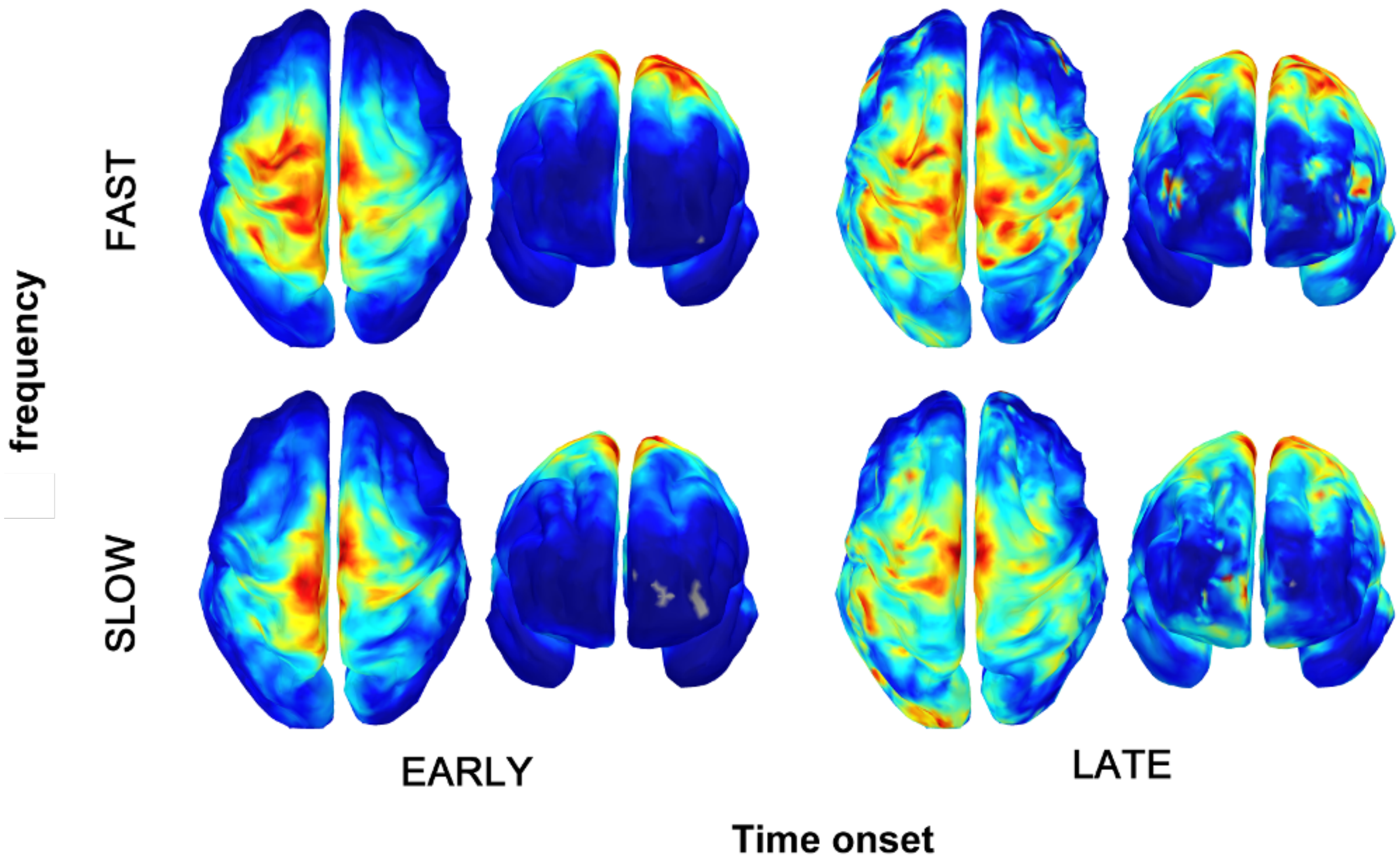

Supplementary Figure 5

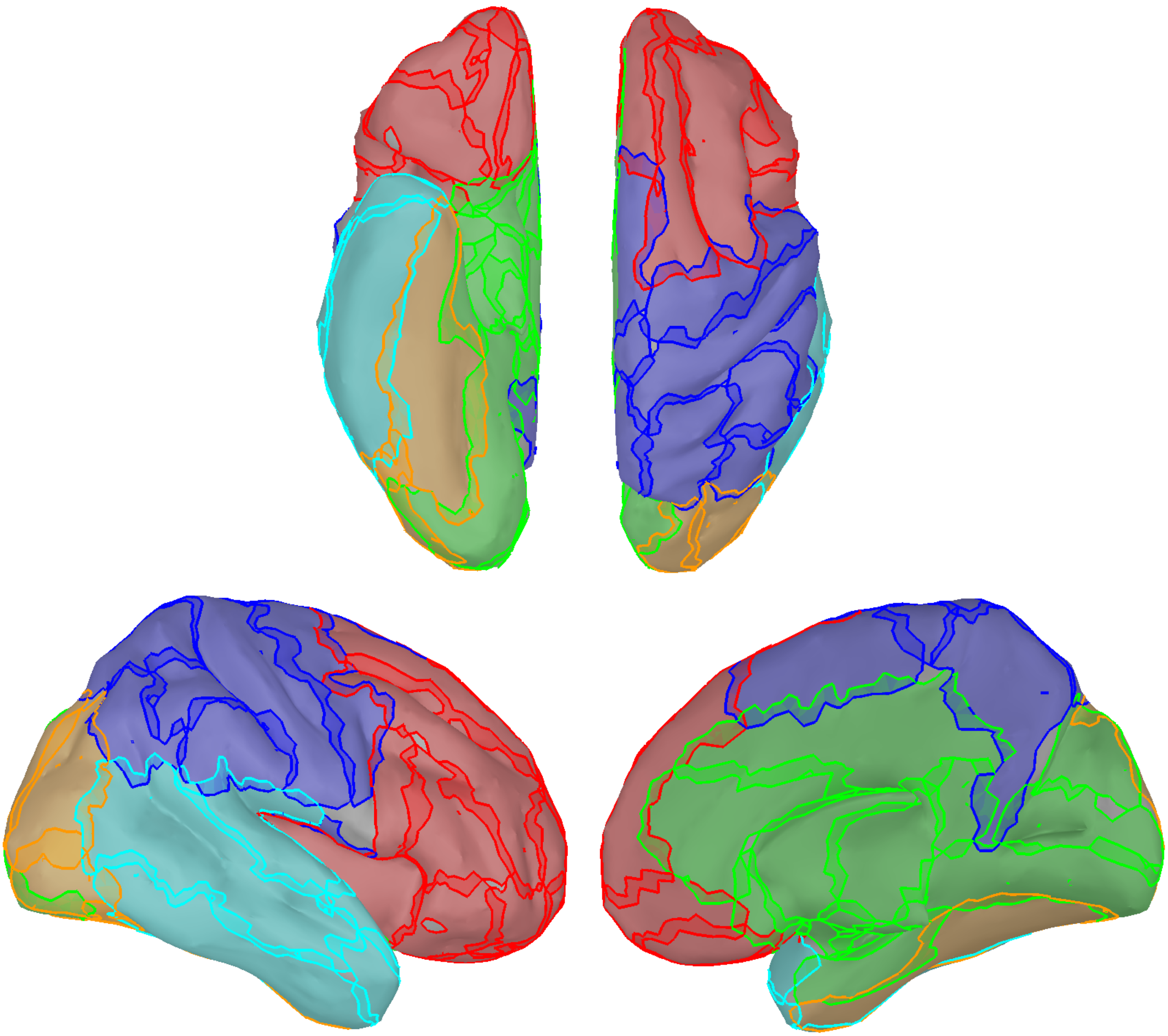

Supplementary Figure 6

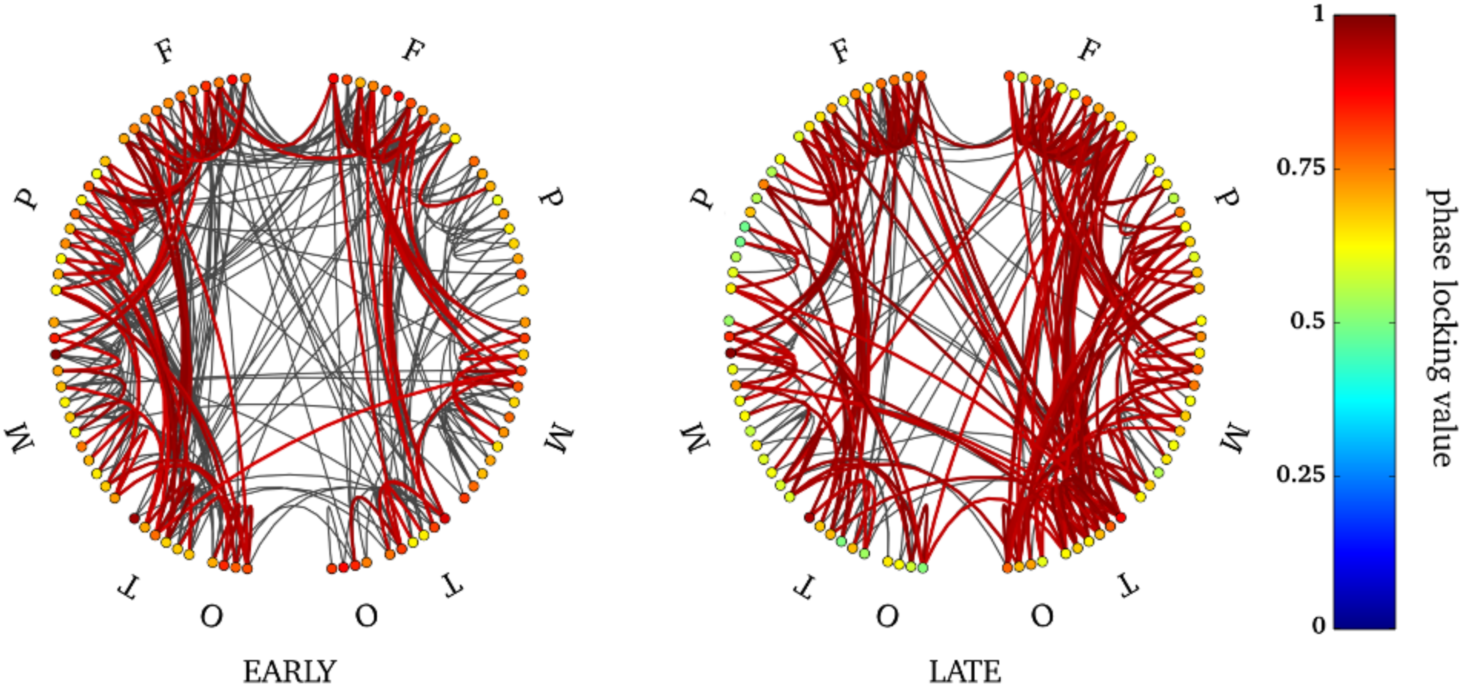

Supplementary Figure 7

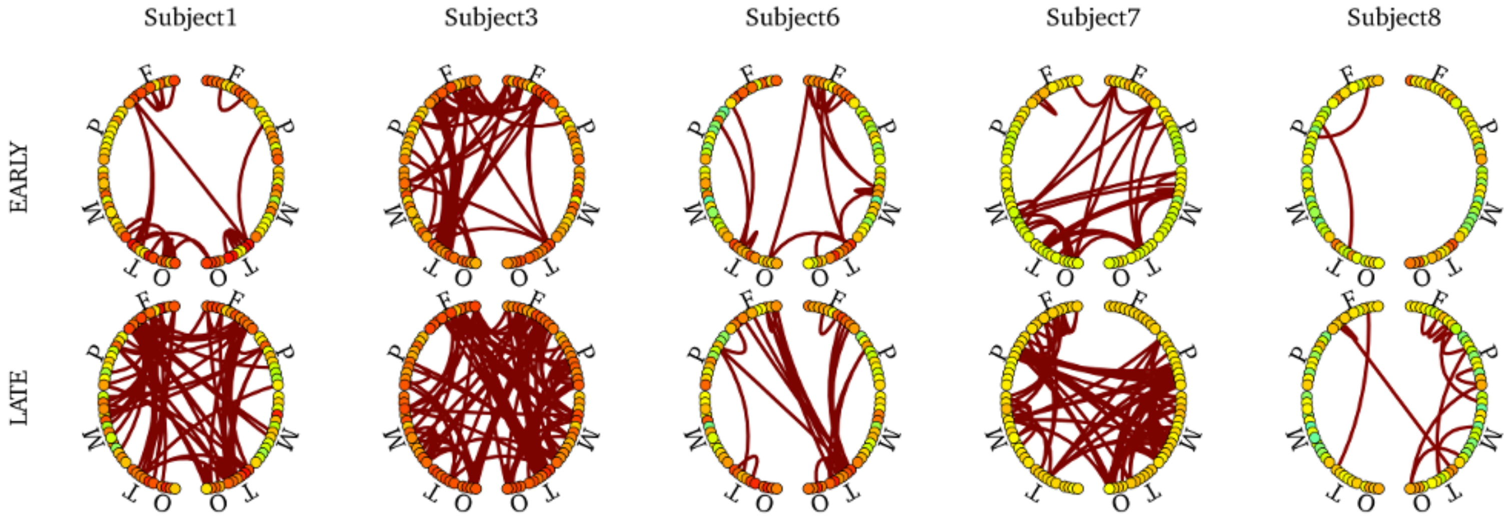

Supplementary Figure 8

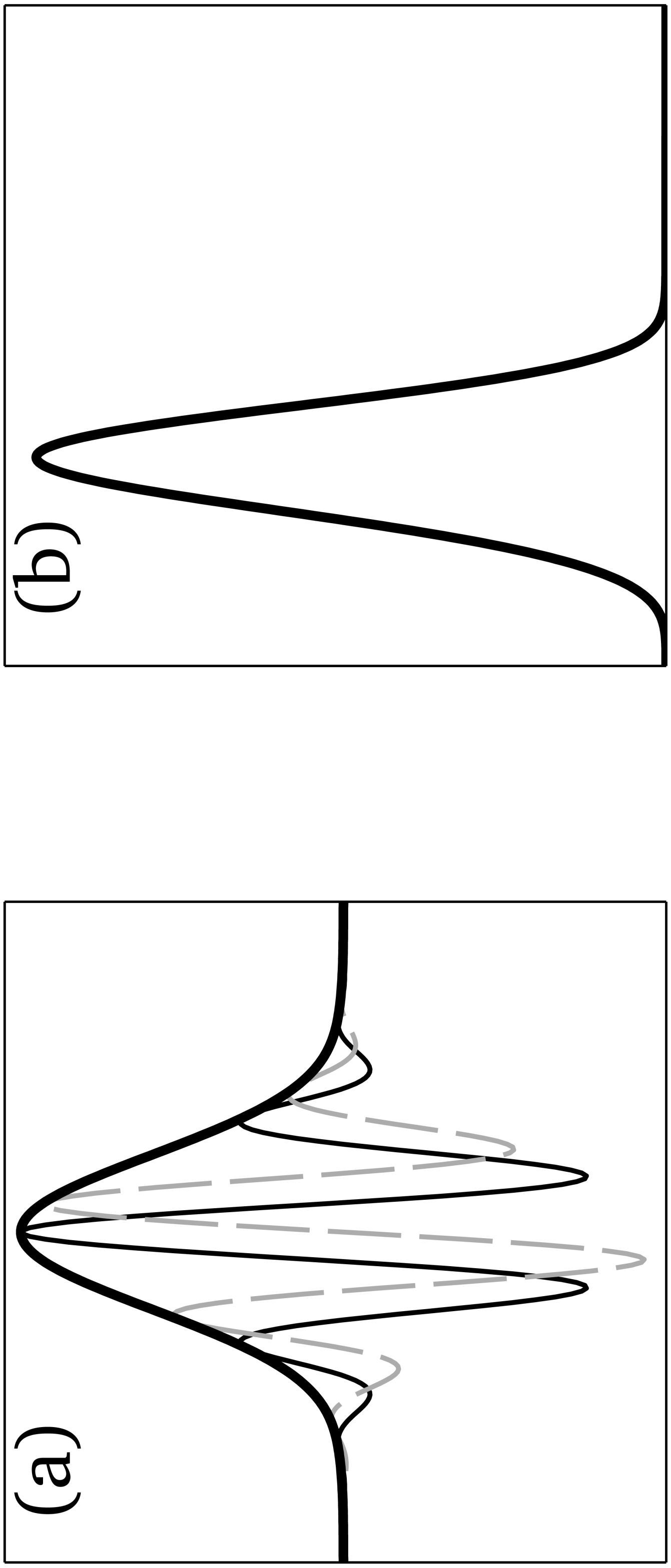

Supplement: Supplementary Figure 1 — Superimposition of the real part of the ridge signal with the original signal of the Figure 1. [file Presentation2.PDF]
